# *Mycobacterium tuberculosis* ClpX Interacts with FtsZ and Interferes with FtsZ Assembly

**DOI:** 10.1371/journal.pone.0011058

**Published:** 2010-07-06

**Authors:** Renata Dziedzic, Manjot Kiran, Przemyslaw Plocinski, Malgorzata Ziolkiewicz, Anna Brzostek, Meredith Moomey, Indumati S. Vadrevu, Jaroslaw Dziadek, Murty Madiraju, Malini Rajagopalan

**Affiliations:** 1 The University of Texas Health Science Center, Tyler Biomedical Research, Tyler, Texas, United States of America; 2 Institute for Medical Biology, Polish Academy of Sciences, Lodz, Poland; University of California Merced, United States of America

## Abstract

FtsZ assembly at the midcell division site in the form of a Z-ring is crucial for initiation of the cell division process in eubacteria. It is largely unknown how this process is regulated in the human pathogen *Mycobacterium tuberculosis.* Here we show that the expression of *clp*X was upregulated upon macrophage infection and exposure to cephalexin antibiotic, the conditions where FtsZ-ring assembly is delayed. Independently, we show using pull-down, solid-phase binding, bacterial two-hybrid and mycobacterial protein fragment complementation assays, that *M. tuberculosis* FtsZ interacts with ClpX, the substrate recognition domain of the ClpXP protease. Incubation of FtsZ with ClpX increased the critical concentration of GTP-dependent polymerization of FtsZ. Immunoblotting revealed that the intracellular ratio of ClpX to FtsZ in wild type *M. tuberculosis* is approximately 1∶2. Overproduction of ClpX increased cell length and modulated the localization of FtsZ at midcell sites; however, intracellular FtsZ levels were unaffected. A ClpX-CFP fusion protein localized to the cell poles and midcell sites and colocalized with the FtsZ-YFP protein. ClpX also interacted with FtsZ mutant proteins defective for binding to and hydrolyzing GTP and possibly for interactions with other proteins. Taken together, our results suggest that *M. tuberculosis* ClpX interacts stoichiometrically with FtsZ protomers, independent of its nucleotide-bound state and negatively regulates FtsZ activities, hence cell division.

## Introduction

*Mycobacterium tuberculosis*, the causative agent of tuberculosis, has spread dramatically worldwide, and recent years have seen not only an increase in the number of multidrug-resistant strains but also the emergence of extensively drug-resistant *M. tuberculosis* [1], [2]. Eradication of *M. tuberculosis* infection necessitates the development of novel drugs targeted to hitherto unidentified metabolic processes and pathways of the pathogen, and FtsZ catalyzed cell division is one such process. FtsZ, the homolog of eukaryotic tubulin, is a highly conserved protein and plays a central and essential role in initiation of the cell division process [3], [4]. *M. tuberculosis* FtsZ, like its bacterial counterparts, exhibits GTP binding and hydrolysis activities and localizes to the midcell division site in the form of a Z-ring [5], [6]. FtsZ-ring assembly in *M. tuberculosis* is delayed under several conditions relevant to its growth; two of these are growth in macrophages and exposure to cephalexin [5]. The identities of the regulators affecting Z-ring assembly and the cell division process in *M. tuberculosis*, however, are largely unknown. Identification and characterization of such regulators would improve our understanding of the cell division process in *M. tuberculosis*.

Elegant genetic and cell biological studies carried out in other bacteria indicate that Z-ring assembly is regulated by the counterbalancing activities of positive and negative regulators that directly or indirectly modulate FtsZ activities (reviewed in [4]). These regulators include FtsA, ZipA, ZapA, Slm, SulA, YneA, Noc, EzrA, CrgA and ClpX; however, not all of these regulators are conserved in all bacteria (reviewed in [7]). The *M. tuberculosis* genome appears to lack most of the known regulators, except for the genes encoding ClpX, CrgA and a YneA-like protein, ChiZ [5], [8]. We recently showed that ChiZ is a DNA damage-inducible protein that shows peptidoglycan hydrolysis activity and functions to regulate Z-ring assembly and cell division in *M. tuberculosis* [5]. It is unknown if ClpX serves as a potential cell division regulator in *M. tuberculosis.* ClpX, the substrate recognition part of the ClpXP protease, is well conserved in prokaryotes [9], [10], [11]. In the ClpXP complex, ClpX is present in a hexameric ring and is attached to the twin-stacked heptameric ClpP rings via the protease interface surface (reviewed [9]). ClpX belongs to the AAA ATPase family and contains the characteristic Walker A and Walker B motifs required for ATP binding and hydrolysis, respectively [12], [13], [14]. By itself, ClpX works as a chaperone, whereas in association with the ClpP protease, it is believed to eliminate misfolded, aggregated and dysfunctional protein targets [9]. It is interesting to note that, unlike *Escherichia coli, Caulobacter crescentus* and *B. subtilis,* the *M. tuberculosis* genome appears to contain one *clp*X and two *clp*P genes, designated as *clpP1* and *clpP2* [15]. On the other hand, the presence of *clpP1* and *clpP2* is common to members of Actinomycetales, including *Mycobacterium spp*. [16], [17].

Recent studies with *C. crescentus and B. subtilis* revealed that ClpX or ClpXP complex functions as a regulator of cell division, although the mechanisms by which it affects cell division appear to vary in the two species. Furthermore, two independent studies with *E. coli* ClpX led to two different conclusions (see below). In the case of Gram negative and alpha-proteobacterium *C. crescentus*, the ClpXP protease regulates the transcription of cell division genes via degradation of the master cell cycle regulator, CtrA [11], [18]. In *E. coli*, another Gram negative bacterium, Camberg et al [19] reported that ClpXP regulates cell division by degrading FtsZ in an ATP-dependent manner, thereby affecting the equilibrium between monomeric and polymeric FtsZ. In contrast, single molecule analysis study with high-speed atomic force microscopy revealed that ClpX regulates dynamics of FtsZ assembly by blocking the reassembly of FtsZ in an ATP independent manner and that ClpXP protease shows only a weak protease activity against FtsZ in the presence of either GTP or GDP [20]. These results, which are inconsistent with the findings of Camberg et al [19], suggest that the contribution of FtsZ unfolding by ClpX is negligible as compared to its role in the inhibition of FtsZ assembly [20]. Nevertheless, the observed intracellular levels of ClpX and FtsZ in *E. coli* appear to be 600 and 15,000 molecules per cell, respectively [21], [22] leading to a suggestion that ClpXP could play a catalytic role in regulating FtsZ assembly. It is pertinent to note that the proteomic studies with *E. coli* lysates utilizing the ClpXP^trap^ protocol identified FtsZ_Ecoli_ as one of the ∼60 proteins associated with ClpXP_Ecoli_ [23]. In *B. subtilis*, ClpX inhibits FtsZ assembly in vivo and interferes with the FtsZ polymerization activity in vitro independent of its ATPase activity [24], [25]. Thus the above divergent results in different organisms necessitate the evaluation of the role of ClpX in cell division in other bacteria including the human pathogen, *M. tuberculosis*.

To begin evaluating the roles of ClpX, if any, in *M. tuberculosis* cell division, we characterized interactions of ClpX with FtsZ in vivo and in vitro. We show that *clpX* expression is elevated during intracellular growth and upon cephalexin treatment, two of the conditions known to delay FtsZ ring assembly [5]. Independently, we show that ClpX functions to regulate FtsZ activity in vitro and FtsZ assembly in vivo by interacting with FtsZ and that both proteins colocalize at the cell division site. Our results are consistent with a model that ClpX activity is one of the factors responsible for the regulation of *M. tuberculosis* Z-ring assembly during intracellular growth and that ClpX action on FtsZ and cell division is possibly conserved in eubacteria.

## Results

### *clp*X expression is upregulated during intracellular growth

**Figure 1:**
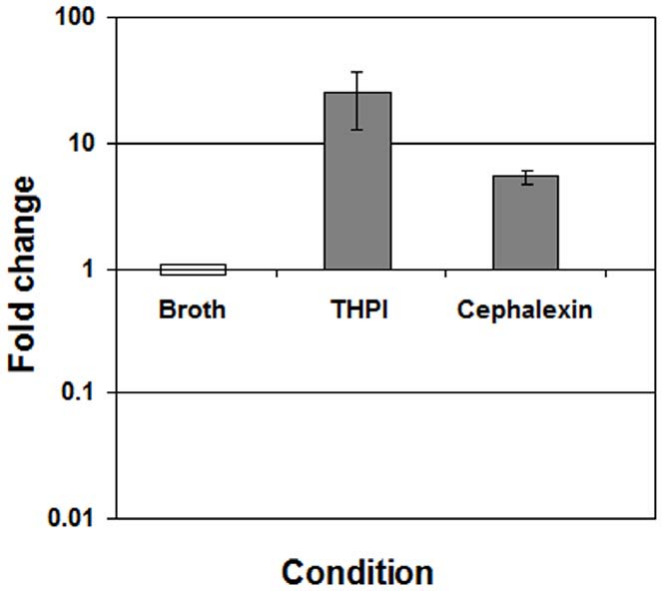
*clpX* expression in *M. tuberculosis*. *M. tuberculosis* cells grown in Middlebrook 7H9 broth with cephalexin and lithium clavulanate (see materials and methods) or in macrophages were used to extract RNA essentially as described [5]. c*lpX* mRNA levels were determined by quantitative real time PCR using TaqMan chemistry. Expression levels of *clpX* were normalized to 16S rRNA and are expressed relative to the *clpX* transcript levels in the broth-grown WT strain. THPI - macrophages from human acute monocytic leukemia cell line. Mean ± SD from three independent RNA samples are shown.

To begin evaluating the role of ClpX in FtsZ catalyzed cell division process in *M. tuberculosis*, we first examined *clp*X expression under select growth conditions where *M. tuberculosis* FtsZ assembly is shown to be modulated, i.e., growth in macrophages and exposure to antibiotics [5], [26]. Accordingly, we determined *clp*X expression relative to the housekeeping gene 16S rRNA by qRT-PCR during intramacrophage growth and upon exposure to cephalexin antibiotic (Fig. 1). Our results indicated that *clp*X expression was upregulated during intramacrophage growth and upon exposure to cephalexin (Fig. 1). Since *M. tuberculosis* cells growing in macrophages and those exposed to cephalexin antibiotic are deficient in FtsZ assembly [5], [26], we examined if ClpX is involved in regulating FtsZ assembly and cell division in *M. tuberculosis*.

### ClpX inhibits the GTP-dependent FtsZ polymerization activity

**Figure 2:**
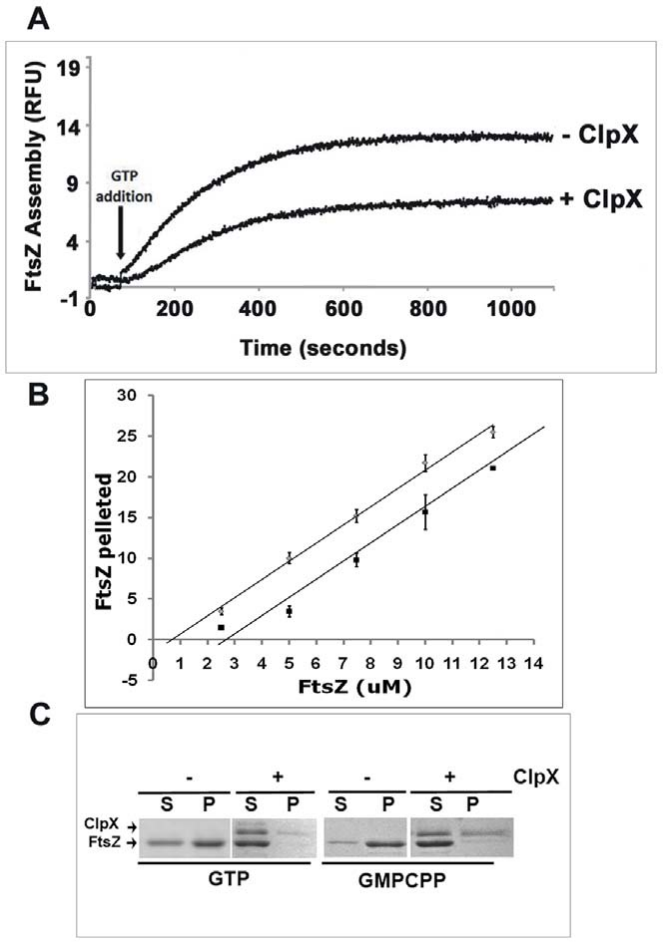
ClpX inhibits the assembly of FtsZ. (**A**) Light scatter assay for FtsZ polymerization in the presence or absence of ClpX. Reactions containing 5.4 µM FtsZ mixed with storage buffer alone or storage buffer containing 2 µM ClpX were initiated with GTP to a final concentration of 1 mM and followed for 15 minutes. Note that both the rate and extent of FtsZ polymerization was decreased in the presence of ClpX. (**B**) Determination of critical concentration of FtsZ polymerization in the presence of ClpX. Reactions containing various concentrations of FtsZ and storage buffer without or with 2 µM ClpX were initiated with 1 mM GTP and sedimentation assays performed as described under Materials and Methods section. The amount of FtsZ polymerized in the absence and presence of ClpX was calculated and plotted to determine the critical concentration of polymerization (see Materials and Methods for details). These assays could not be carried out at higher concentrations of ClpX as our innumerable attempts to obtain concentrated stocks of active ClpX following the described refolding protocol were not successful (see Materials and Methods). (**C**) Effect of non-hydrolysable GTP analog, GMPCPP on FtsZ polymerization activity [30], [31]. ClpX inhibition of FtsZ assembly was examined in the absence of GTPase activity using GMPCPP. FtsZ (2.3 µM) without or with ClpX (2 µM) was incubated in the presence of either 1 mM GMPCPP or GTP. FtsZ polymers were collected by centrifugation, supernatant (lanes marked as ‘S’) and pellet (lanes marked as ‘P’) fractions were loaded on SDS-PA and visualized by coomassie staining.

The 90° right angle light scatter assay is a widely used method to measure the GTP-dependent polymerization of FtsZ [27]. To examine whether ClpX interferes with FtsZ polymerization activity, purified FtsZ (5.4 µM) was incubated without or with ClpX (2 µM) at 30°C and polymerization was initiated with 1 mM GTP. Since ClpX is thought to possess ATPase activity, 1 mM ATP was included in all polymerization reactions. Consistent with our earlier data, the presence of ATP had no effect on GTP-dependent FtsZ polymerization (data not shown; [28]). In the presence of ClpX, an ∼35% reduction in GTP-dependent FtsZ assembly was noted (Fig. 2A). Control experiments with RecA, an ATPase (reviewed in [29]), (Supplementary data Fig. S1) or BSA (data not shown) had no effect on FtsZ polymerization. These results suggest that *M. tuberculosis* ClpX interferes with FtsZ polymerization.

To gain insights into the mechanism by which ClpX inhibits FtsZ assembly, increasing concentrations of FtsZ were incubated with a fixed concentration of ClpX (2 µM) and sedimentation assays performed to determine the extent of FtsZ polymerization (supplementary Fig. S2, panel B). A control experiment lacking ClpX was also performed (supplementary Fig. S2, panel A). As can be seen, in the absence of ClpX, FtsZ polymers were present in the pellet at 1 µM and beyond, (supplementary Fig. S2, panel A, compare lanes S with P). Determination of the amount of FtsZ polymer in the pellet as a function of FtsZ concentration revealed that the critical concentration for FtsZ polymerization was ∼1 µM. In the presence of 2 µM ClpX, the amount of FtsZ in the pellet at all protein concentrations was decreased (supplementary Fig. S2, panel B). Furthermore, densitometric determination of the FtsZ levels in the supernatant and pellet fractions revealed that irrespective of the concentration of FtsZ used, the amount of FtsZ in the supernatant was the same, and the critical concentration for FtsZ polymerization was increased to ∼2.4 µM (Fig. 2B). Owing to the difficulty in purifying higher than ∼8 µM ClpX, these experiments could not be performed at higher concentrations of ClpX. Nonetheless, the above data indicate a stoichiometric association between the FtsZ and ClpX molecules and suggest that ClpX modulates FtsZ assembly by sequestration.

In the above experiments, ClpX was added along with FtsZ. Next, we added ClpX to preformed FtsZ polymers (supplementary Fig. S3). Even under these conditions, unlike with the buffer control, FtsZ was present in the supernatant (supplementary Fig. S3, see reactions 4 and 5, and compare with 2 and 3). Together, these experiments indicate that ClpX interferes with FtsZ polymerization regardless of whether it is added before the initiation of polymerization or after the formation of FtsZ polymers.

### GTPase activity of FtsZ is not needed for inhibition of polymerization by ClpX

We next examined the inhibition of FtsZ_TB_ assembly by ClpX in the presence of GTP or GMPCPP, a non-hydrolyzable analog of GTP (Fig. 2C) [30], [31]. FtsZ_TB_ polymerization occurred in the presence of GMPCPP, as with GTP. Addition of ClpX interfered with the assembly of FtsZ_TB_ in the presence GMPCPP (Fig. 2C). These data indicate that ClpX inhibition of FtsZ assembly is independent of the GTP hydrolysis activity of FtsZ.

### FtsZ interacts with ClpX

The above data are consistent with the idea that the ClpX-mediated interference with *M. tuberculosis* FtsZ polymerization activity involves direct physical interaction between the two proteins in *M. tuberculosis*. Although *M. tuberculosis* FtsZ exhibits GTP binding and hydrolysis activities like other bacterial FtsZ counterparts, notable differences in these properties have been observed [28], [32]. Furthermore, as reviewed, the *M. tuberculosis* genome lacks several identifiable homologs of known proteins of the bacterial cell division machinery [5], [33], [34]. Hence, we evaluated physical and functional interactions between the ClpX and FtsZ proteins of *M. tuberculosis* using a variety of in vitro and in vivo assays.

#### In vitro: Pull-down assay shows that FtsZ copurifies with His-ClpX

**Figure 3:**
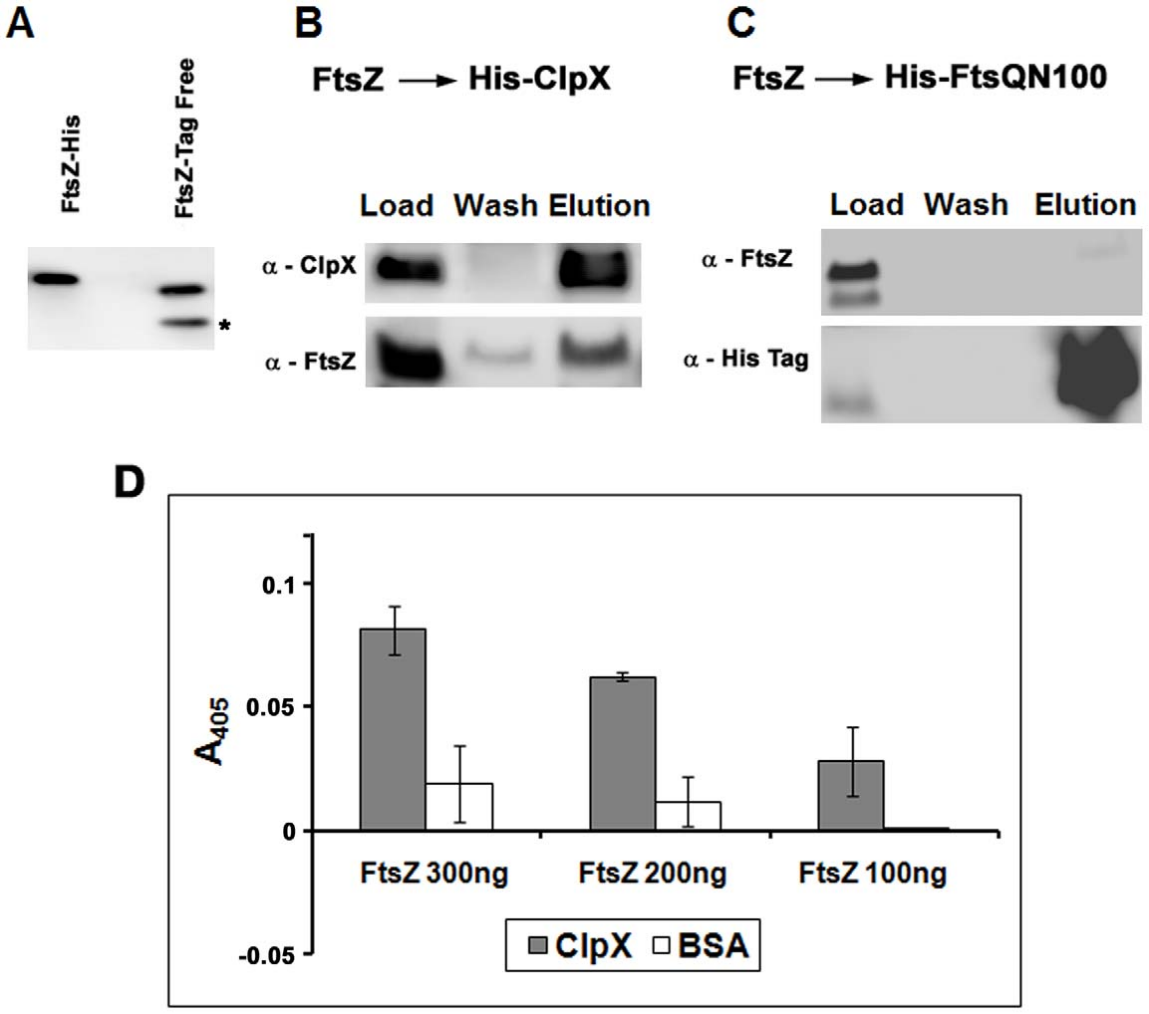
FtsZ interacts with ClpX in vitro. (**A**) Visualization of FtsZ-His and tag-free FtsZ on a NuPAGE gel. Note that the mobilities of both proteins are distinct. *Digestion of His-FtsZ with thrombin to remove the polyhistidine tag often resulted in non-specific cleavage at another site in FtsZ resulting in a truncated FtsZ protein. However, this cleavage did not interfere with the interactions of FtsZ with ClpX. (**B**) Immunoblots of FtsZ pulled down with His-ClpX on Ni-NTA resin. Equimolar amounts of purified FtsZ and His-ClpX were mixed and loaded onto Ni-NTA resin. Following washing, bound proteins were eluted with 0.3 M imidazole and resolved on a 10% NuPAGE gel, transferred to PVDF membrane and probed with α-FtsZ or α-ClpX_BS_ antibodies. Load, wash and elution are shown. (**C**) FtsZ does not copurify with FtsQΔN100. FtsZ was mixed with His-FtsQΔN100 and loaded on Ni-NTA resin. Proteins eluted with 0.3 M imidazole were resolved on a 10% NuPAGE gel, transferred to a PVDF membrane and probed with α-FtsZ or α-His antibodies. Load, wash and elution are shown (**D**) Solid-phase binding assay for FtsZ with ClpX. Wells of a microtiter plate were coated with ClpX, or BSA and incubated with various concentrations of FtsZ protein as indicated. The bound FtsZ protein was immunodetected with α-FtsZ antibodies and ELISA (AnaSpec), as described in the methods section. Mean ± SD from three independent experiments are shown.

A pull-down assay was performed to evaluate interactions between the ClpX and FtsZ proteins. Equimolar amounts of purified recombinant His-ClpX protein (molarity based on monomer concentration) and a tag-free FtsZ protein created by cleaving the polyhistidine tag were mixed and applied to Ni-NTA resin. Following washing, bound proteins were eluted with imidazole and detected by immunoblotting using anti-ClpX_BS_ and anti-FtsZ antibodies. Both His-FtsZ and tag-free FtsZ showed distinct mobilities on NuPAGE gels (Fig. 3A). As can be seen, the elution fraction containing ClpX also contained FtsZ (Fig. 3B). Some FtsZ was also found in the flow-through and early wash fractions (data not shown), indicating that not all of the FtsZ was complexed with ClpX. In a reverse experiment, we were able to pull-down MBP-ClpX with His-FtsZ on Ni-NTA resin (data not shown). Salt concentrations of 0.2 M weakened the ClpX-FtsZ complexes and those of 0.5 M nearly abolished the interaction (Supplementary Fig. S4, panel A). ClpX-FtsZ complexes were also isolated when *E. coli* lysates containing the His-ClpX and FtsZ-S-tag were processed on nickel-affinity columns (Supplementary Fig. S4, panel B). Control experiments with lysates containing recombinant N-terminal FtsQ protein (amino acid 1–100; Table S2), His-FtsQN100, and FtsZ did not recover FtsZ in the fractions containing His-FtsQN100 (Fig. 3C), indicating that the observed interaction between ClpX and FtsZ is specific.

#### In vitro: Solid-phase binding assay confirms ClpX-FtsZ interactions

Next, a solid-phase binding assay was carried out to further confirm interactions between FtsZ and ClpX. In this assay, ClpX or BSA immobilized in the wells of a microtiter plate was incubated with increasing concentrations of FtsZ. Following washing, the bound FtsZ protein was immunodetected and quantified by ELISA. As can be seen, FtsZ bound to the ClpX-coated wells but not to the wells coated with BSA (Fig. 3D).

#### In vivo: BACTH assays reveal interactions between ClpX and FtsZ

**Figure 4 pone-0011058-g004:**
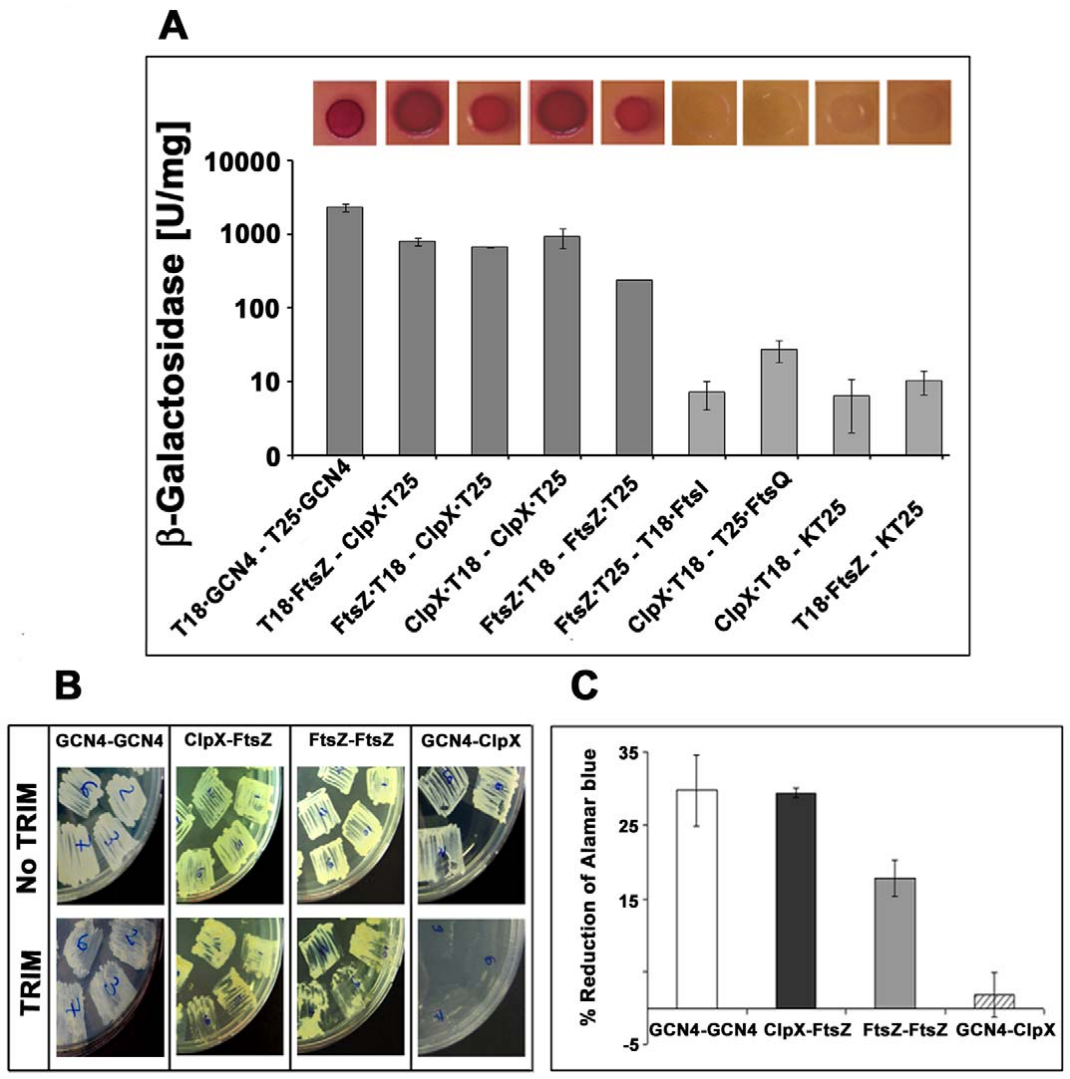
FtsZ interacts with ClpX in vivo. (**A**) BACTH assays showing FtsZ-ClpX interactions. *clpX*, *ftsZ, ftsI and ftsQ* fusions to T18 or T25 fragments of adenylate cyclase were cloned into various BACTH vectors and interactions were examined as described [35]. *E. coli* BTH101 recombinants bearing indicated combinations of plasmids were plated on MacConkey agar containing 1% maltose. Red colonies indicate strong positive interactions (Inset). FtsZ-FtsZ and GCN4-GCN4 served as positive controls and ClpX-FtsQ, FtsZ-FtsI, served as negative controls. β-galactosidase activity of the indicated recombinant strains was measured and is shown. Assay methodology is as described in the text. Mean ± SD values from three independent experiments are shown. (**B**) M-PFC assay for detecting the interactions between FtsZ and ClpX proteins in *M. smegmatis*. Recombinant *M. smegmatis* strains producing FtsZ and ClpX fused to DHFR1, 2 or DHFR3 were grown on 7H11/Km/Hyg or 7H11/Km/Hyg/Trim. Growth on Trim plates indicates strong positive protein-protein interaction. (**C**) Quantitation of M-PFC interactions using fluorescent alamar blue dye. *M. smegmatis* strains coexpressing the tested interaction pairs were propagated in 7H9 broth with 17.85 µg/ml trimethoprim and viability was determined using the fluorescent alamar blue assay [37]. Color change of alamar blue from non-fluorescent blue to a fluorescent pink indicates positive protein-protein interaction. Note GCN4[F1,2]/GCN4[F3] is a positive control whereas ClpX[F1,2]/GCN4[3] is negative control. The vector control strain DHFR[1], [2]/DHFR[3] was used for background subtraction. Samples were analyzed in duplicate and mean ± SD from two experiments are shown.

Next, BACTH assays were performed to evaluate in vivo interactions between ClpX and FtsZ [35]. In these assays, *fts*Z and *clp*X genes were cloned as fusions to the T25 or T18 fragments of adenylate cyclase in two separate vectors and transformed into the *E. coli* reporter strain, BTH101 [35]. Functional complementation of adenylate cyclase activity due to interactions between the partners leads to cAMP production and subsequent transcription of the *lac* reporter gene, which gives a distinct color to colonies growing on indicator agar plates. The strength of the interaction was also measured by assaying for the β-galactosidase activity. As expected, the *gcn4-gcn4* positive control strain showed high β-galactosidase activity (Fig. 4A). Transformants expressing *ftsZ* or *clpX* from both vectors were also red, indicating self-interactions in these proteins (Fig. 4A). Similarly, the transformants expressing *ftsZ* and *clp*X were also red, but not when *clpX* or *ftsZ* was expressed with the control plasmid (see Fig. 4A). The amount of β-galactosidase produced with the transformants expressing *fts*Z and *clp*X was comparable to the *gcn4-gcn4* positive control and also to those expressing *clp*X or *fts*Z from both vectors indicating that the interaction between ClpX and FtsZ is strong (Fig. 4A). These results were consistently obtained with any combination of vectors used to express the *clp*X and *fts*Z genes (Fig. 4A). The specificity of ClpX-FtsZ interaction was indicated by the lack of interaction between ClpX and FtsQ and also between FtsZ and FtsI (see [36]) (Fig. 4A). Interactions between ClpXΔN200, lacking the N-terminal Zinc-binding, ATP binding and ATP hydrolysis domains, and FtsZ appeared to be weak, as the amount of β-galactosidase produced was just above the background. These data suggest that the N-terminal region of ClpX is important for optimal interactions with FtsZ (supplementary Fig. S5, panel A; Table S2). Together, the above results validate the in vitro data showing interactions between the ClpX and FtsZ proteins.

#### In vivo: M-PFC assay confirms ClpX and FtsZ interactions

We next examined the interactions between ClpX and FtsZ proteins in their native environment using the M-PFC assay [37]. This assay scores for trimethoprim (Trim) resistance due to the regeneration of functional murine dihydrofolate reductase (mDHFR) activity from two independent mDHFR protein fragment domains fused to two potential protein interaction partners. The strength of interaction is measured by monitoring the reduction of alamar blue in growth media containing Trim [37]. Accordingly, we fused full-length *clp*X and *fts*Z to the 3′ or 5′ end of murine *dhfr* fragments *1–2* and *3* in bait and prey vectors, respectively, and expressed them from the tetracycline-inducible promoter in *M. smegmatis* (see methodology section and Table S2). The recombinant strains expressing *Ptet::clpX-dhfr1,2* and *Ptet::ftsZ-dhfr3* showed resistance to Trim (Fig. 4B), similar to those expressing *ftsZ*/*fts*Z or *gcn*4/*gcn*4, the two positive controls. Negative control transformants expressing *gcn*4 and *clp*X did not show any growth on Trim plates (Fig. 4B). Evaluation of the extent of alamar blue reduction confirmed these results (Fig. 4C). Transformants expressing *Ptet::clpX*Δ*N200-dhfr1,2* and *Ptet::ftsZ-dhfr3* did not grow on Trim plates (Supplementary Fig. S5, panel B) corroborating the conclusion from BACTH data that the N-terminal 200 amino acids of ClpX are needed for interaction with FtsZ.

### ClpX interaction with mutant FtsZ proteins

**Figure 5:**
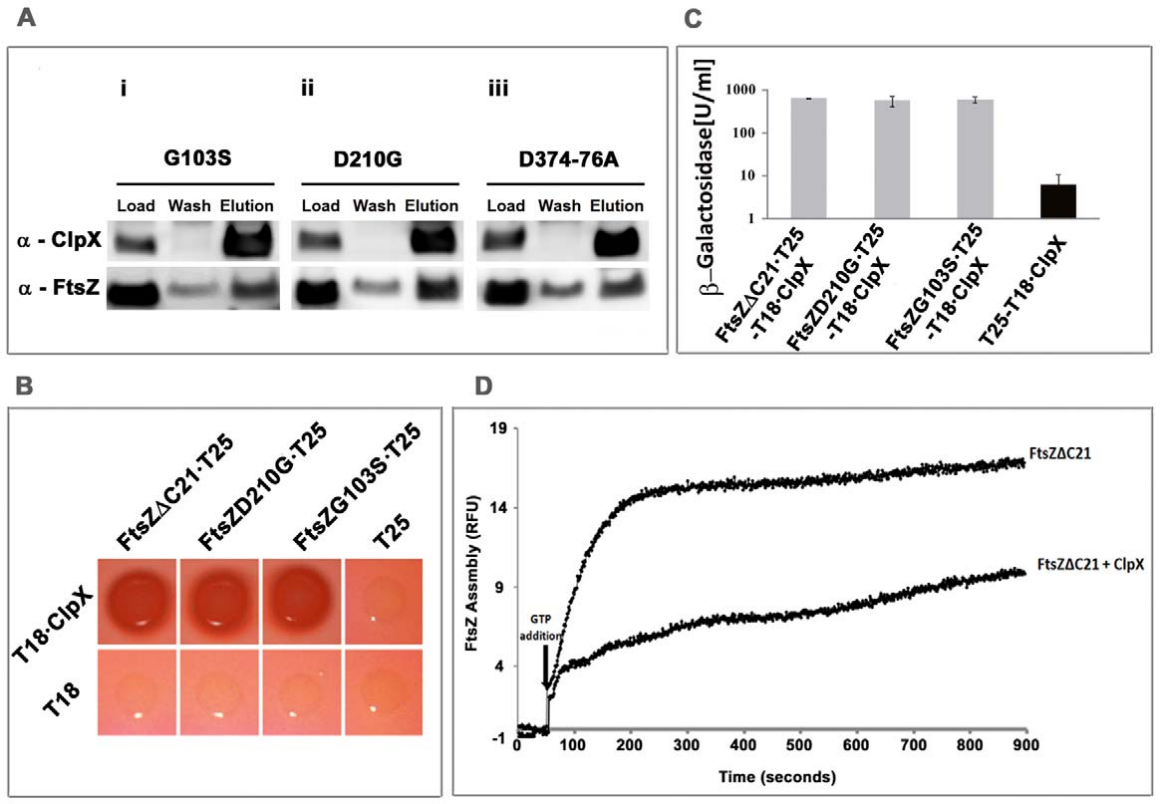
ClpX interacts with various FtsZ mutants. (**A**) **Pull-down assay.** Immunoblots of His-ClpX and various tag-free FtsZ mutant proteins copurified on Ni-NTA resin. Equimolar amounts of ClpX and various FtsZ mutant proteins were used for the pull-down assay. Load, wash and proteins eluted with 0.3 M imidazole were resolved on a 10% NuPAGE gel, transferred to a PVDF membrane and probed with α-ClpX_BS_ and α-FtsZ antibodies. (i) FtsZ_G103S_, (ii) FtsZ_D210G_ and (iii) FtsZ_D374-76A_. (**B**) Bacterial two-hybrid analyses of ClpX-FtsZ mutant protein interactions. Experimental details are as in Fig. 4A. Interactions of ClpX-FtsZΔC21, ClpX-FtsZ_G103S_, and ClpX-FtsZ_D210G_ on MacConkey agar plates containing 1% maltose. Red indicates strong interaction. (**C**) Quantitative analysis of BACTH interactions: Extent of interactions was measured by β-galactosidase activity as described under Fig. 4A. Mean ± SD from three experiments are shown. (**D**) ClpX inhibits polymerization of FtsZΔC21. The effect of ClpX on FtsZΔC21 assembly was monitored by right angle light scatter assay. ClpX (2 µM) was mixed with FtsZΔC21 (5. 4 µM) in the presence of GTP at a final concentration 1 mM. Note both the extent and the rate of FtsZΔC21polymerization was decreased in the presence of ClpX.

As reviewed, FtsZ binds GTP and exhibits GTP-dependent polymerization and GTP hydrolysis activities [28], [32]. FtsZ also interacts with FtsW in vitro [38] and in vivo [34]. The amino acid residues in FtsZ important for these activities have been identified. For example, FtsZ_G103S_ is defective in binding to GTP; hence, is also defective in GTP hydrolysis. FtsZ_D210G_, on the other hand, is proficient in GTP binding, but is defective in GTP hydrolysis [28]. FtsZ_D374-76A_ is defective in its interactions with FtsW [34]. FtsZ_ΔC21_ lacks 21 amino acids from the C-terminus, a region believed to be critical for interactions of FtsZ with other cell division proteins [38], [39], [40], [41]. To investigate if ClpX interacts with FtsZ mutants defective in the above-described activities, we purified FtsZ_G103S_, FtsZ_D210G_, FtsZ_D374-76A_ and FtsZ_ΔC21_ recombinant proteins and investigated their interaction using pull-down and BACTH assays [34], [35], [38]. Pull-down assays with mixtures of equimolar amounts of His-ClpX and various FtsZ mutant proteins followed by immunoblotting confirmed the presence of FtsZ in fractions containing ClpX (Fig. 5A). The BACTH assay confirmed these data (Fig. 5B, 5C). ClpX also inhibited the assembly of FtsZΔC21 (Fig. 5D). These data suggest that ClpX is capable of interacting with monomeric or polymeric FtsZ and that the C-terminal region of FtsZ is not required for this interaction.

### Overexpression of *clpX* inhibits Z-ring assembly and reduces *M. tuberculosis* viability

**Figure 6:**
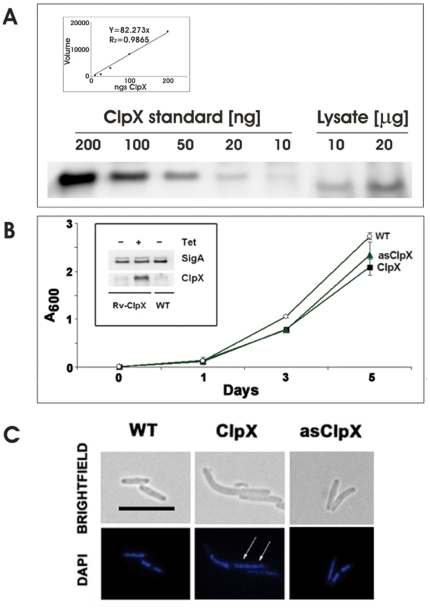
Overproduction of ClpX delays growth and cell division of *M. tuberculosis*. (**A**) **Intracellular levels of ClpX in WT** ***M. tuberculosis:*** Ten and twenty micrograms of clear protein lysates from WT *M. tuberculosis* were resolved on a 10% NuPAGE gel, transferred to a PVDF membrane and probed with α-ClpX_BS_ antibodies. Various known concentrations of recombinant ClpX were also resolved on the same gel and processed under the same conditions. ClpX bands were quantitated by volumetric analysis using QuantityOne software. A standard curve shown in the inset is prepared from pure protein standards and was used to determine the amount of ClpX in the lysates (see materials and methods for details). (**B**) Growth of *M. tuberculosis clpX* strains. *M. tuberculosis* strains with elevated (ClpX) or decreased ClpX levels (asClpX) were grown in broth in the presence of 100 ng/ml anhydrotetracycline. The *M. tuberculosis* H37Rv strain served as the control strain. Growth was monitored by absorbance at 600 nm. Mean ± SD from three independent experiments are shown. **Inset** - ClpX levels in *M. tuberculosis clpX* strains. Cellular lysates (8 µg) were resolved on a 10% NuPAGE gel, transferred to a PVDF membrane and probed with α-ClpX or α-SigA antibodies. Bands were quantified using QuantityOne Software in a BioRad Imager. ClpX levels were normalized to SigA and fold change with respect to WT strain was determined (see text). (**C**) Effect of altered ClpX levels on *M. tuberculosis* cell and nucleoid morphology. Exponential cultures of ClpX sense and antisense *M. tuberculosis* strains were induced with 100 ng/ml anhydrotetracycline for 48 hrs. Cells were stained with DAPI as described in the text and examined by fluorescence microscopy. WT - wild type, ClpX - ClpX overproducing strain and asClpX - ClpX antisense strain. Images were optimized using Adobe Photoshop 7.0. Bar - 5 µm. Arrows - multiple nucleoids.

Saturated transposon mutagenesis studies have indicated that *clp*X is an essential gene, like the *fts*H protease [42]. While it is still possible that *clpX* is not essential in *M. tuberculosis*, saturation transposon screens are comprehensive. We would like to note that our innumerable attempts to make a knock-out *ftsH*, another protease shown by transposon mutagenesis to be essential, by homologous recombination were unsuccessful (Chauhan and Rajagopalan, unpublished data). Therefore, to evaluate the consequences of the interaction of ClpX with FtsZ in vivo, we created *M. tuberculosis* strains expressing altered levels of ClpX from the inducible tetracycline promoter and characterized them with respect to growth, viability and Z-ring structures. Quantitative immunoblotting determined the intracellular levels of ClpX monomers in exponential phase cultures of WT *M. tuberculosis* to be ∼14,000 molecules per cell (Fig. 6A, see legend for details). Under the same conditions, FtsZ levels were previously determined to be ∼30,000 molecules per cell (12), and this makes the approximate ratio of ClpX (monomeric) to FtsZ 1∶2. Immunoblotting also revealed that the ClpX levels were elevated by ∼six-fold in the presence of anhydrotetracycline in the sense *clpX* strain (pRD23, Table S2; inset in Fig. 6B). The low level of the ClpX signal in the antisense strain (asClpX) made it difficult to accurately determine the reduction in ClpX levels in this strain (data not shown). As an alternative, we evaluated *clp*X transcript expression by qRT-PCR (supplementary Fig. S6). The *clp*X transcript expression relative to the housekeeping gene 16S rRNA was decreased by 12-fold in the asClpX strain (supplementary Fig. S6).

We next examined if intracellular levels of FtsZ were altered in cells producing altered levels of ClpX. The FtsZ levels were neither decreased under ClpX overproduction condition nor increased under ClpX underproduction condition, i.e. in as*Clp*X strain (supplementary Fig. S7, panels A and B). Together, these results suggest that altered levels of ClpX do not affect intracellular *M. tuberculosis* FtsZ levels. We also found that *M. tuberculosis* sense and antisense *clp*X strains showed a moderate reduction in growth (Fig. 6B) and viability (supplementary Fig. S8). These defects can possibly be attributed to interruptions in the ClpX chaperone and protein degradation functions due to reduction in ClpX levels.

The merodiploids overexpressing *clp*X were elongated, with an ∼50% increase in cell length (3.3 µm ±0.25) as compared to WT (2.2 µm ±0.58; Fig. 6C, compare brightfield panels WT and ClpX). Visualization of nucleoid staining with DAPI revealed elongated cells with multiple nucleoids (Fig. 6C, see arrows in ClpX panels). In contrast, the WT cells had one or two distinct nucleoids per cell (Fig. 6C, WT panels). A decrease in ClpX levels caused only a modest 7% increase in cell length (2.36 µm ±0.11), and the DAPI staining patterns were similar to WT (Fig. 6C, asClpX panels).

**Figure 7:**
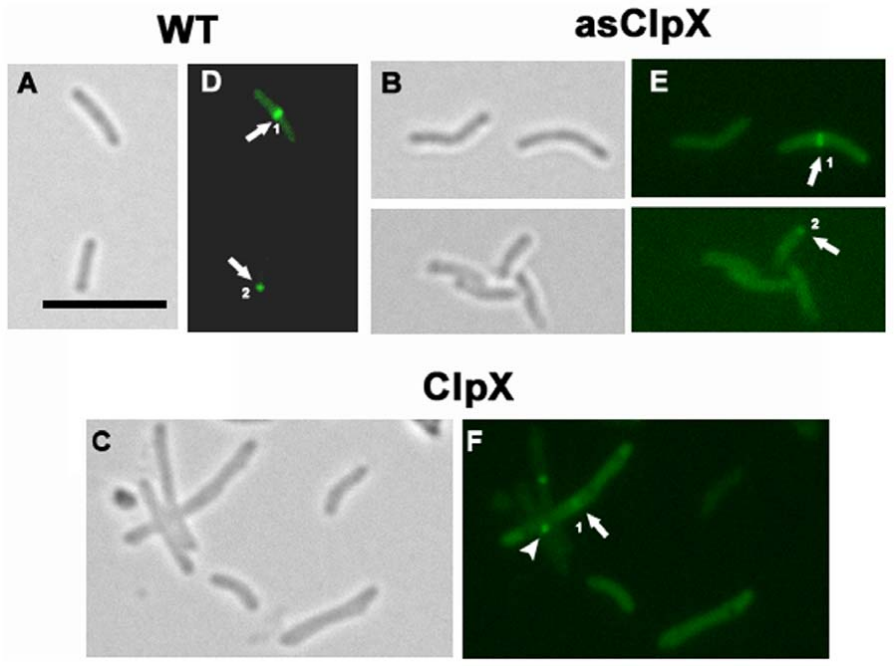
Altered ClpX levels affect FtsZ ring formation in *M. tuberculosis*. *M. tuberculosis Ptet::ftsZ-gfp* strains producing either elevated (*Ptet::clpX*) (ClpX) or decreased (asClpX) (*Ptet::asclpX*) levels of ClpX were examined by fluorescence microscopy. Control strain is *M. tuberculosis Ptet::ftsZ-gfp* (WT). These strains were induced with 5 ng/ml anhydrotetracycline for 24 h and cells were visualized by microscopy. A, B, and C are brightfield images and D, E, and F are the corresponding fluorescence images. Arrows and arrowheads represent FtsZ-GFP structures. Arrow 1 and arrow 2 represent midcell and polar localizations, respectively, whereas arrowhead shows aberrant localization. Bar - 5 µm.

To determine whether the observed morphological changes associated with ClpX overproduction were due to defects in FtsZ ring assembly, we created an *M. tuberculosis* strain expressing *Ptet::ftsZ-gfp* and *Ptet::clpX*, visualized FtsZ structures and compared the results with the control strain expressing *Ptet::ftsZ-gfp.* Incubation of the control strain with 5 ng of anhydrotetracycline for 24 h revealed distinct bright FtsZ-structures at the midcell position (Fig. 7D, see arrow 1) and in some cases, at the cell poles (Fig. 7D, arrow 2). The midcell rings accounted for 16.8±1.4% of cells (n = 627). The polar FtsZ localization, believed to be a remnant from the previous cell division, accounted for approximately 6.5% of cells. These results are consistent with our earlier published data [26]. In contrast, *M. tuberculosis* cells expressing *Ptet::fts*Z-*gfp* and *Ptet::clp*X were elongated, with an average cell length of 5.5±1.0 µms (Fig. 7, compare panel A with C). Unlike the WT cells (2.1±0.6 µms), many elongated cells were devoid of bright Z-rings and had diffuse fluorescence along the entire cell length. Approximately 10.8±2.2% of cells had less-fluorescent midcell Z-rings, which tended to bleach quickly (see discussion). Polar FtsZ structures were rarely present (Fig. 7, panel F), and some elongated cells had aberrant FtsZ-GFP localization (Fig. 7, panel F, see arrowhead). The average length of FtsZ-GFP ring containing cells increased by 52% under ClpX overproduction conditions. Increased cell length and diffuse midcell Z-rings are consistent with the idea that increased ClpX levels delay FtsZ assembly and cell division in *M. tuberculosis*.

The antisense *clp*X cells showed FtsZ structures at midcell sites and cell poles, as in the WT strain, but these structures were less vibrant (Fig. 7, asClpX panels). Since all strains were grown and processed under similar conditions, we believe that the diffuse fluorescence of FtsZ-GFP rings in asClpX strain is not an artifact of microscopy. Given that *clpX* is an essential gene [42], lowered ClpX levels might therefore affect the activities of other proteins and/or other potential regulators of cell division.

### ClpX-CFP localizes to the cell poles and to midcell sites and colocalizes with FtsZ

**Figure 8:**
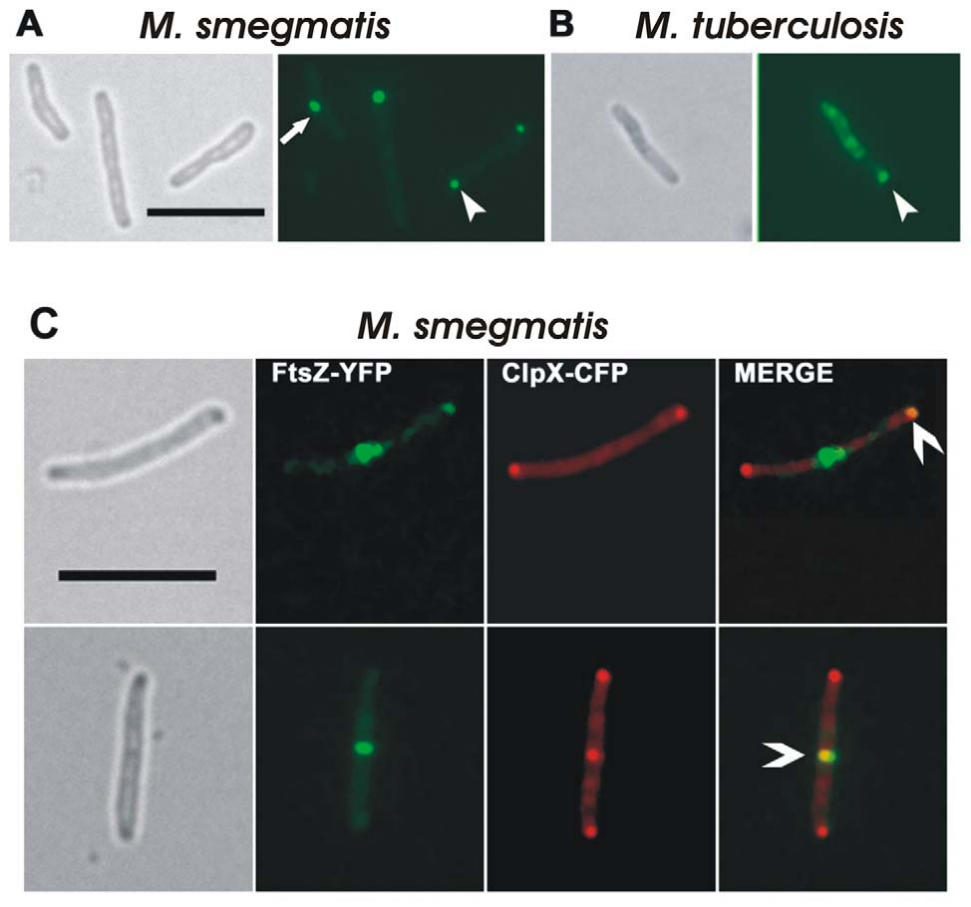
Localization of ClpX and FtsZ proteins in *M. smegmatis* and *M. tuberculosis*. (**A**) *M. smegmatis*, (**B**) *M. tuberculosis*. Recombinant mycobacterial strains producing ClpX-CFP were propagated in broth with 5 ng/ml anydrotetracycline for 1 h *(M. smegmatis)* or 24 h (*M. tuberculosis).* Brightfield and respective fluorescence images are shown. Arrow - midcell localization of ClpX; arrowhead - polar localization. (**C**) Colocalization of ClpX and FtsZ. *M. smegmatis* expressing *Pami::ftsZ-gfp* and *Ptet::clpX-cfp* was grown in broth containing 5 ng/ml anydrotetracycline and acetamide at a final concentration of 0.2% for 1 h. The levels of ClpX-CFP and FtsZ-YFP induced under these conditions did not lead to overproduction associated phenotypes. Individual brightfield and fluorescent images are shown. Bar - 5 µm. FtsZ-YFP - pseuocolored green, ClpX-CFP - pseudocolored red and their colocalization as yellow spots. Split arrow - colocalization of ClpX and FtsZ proteins.

Visualization of fewer and fainter FtsZ-structures under ClpX overproduction conditions suggested that fewer FtsZ molecules were in the midcell Z-ring. This could be due to sequestration of FtsZ protomers by ClpX thereby limiting the available pools of FtsZ for polymerization. Alternatively, ClpX association with FtsZ in the midcell Z-rings could lead to the latter's disassembly. Both possibilities are not mutually exclusive. To address the latter possibility, we attempted to visualize ClpX structures by creating an *M. tuberculosis* strain expressing *Ptet::clpX-cfp* and determining whether ClpX colocalizes with FtsZ. The ClpX-CFP structures in *M. tuberculosis* were less distinct and generally more diffuse (Fig. 8, panel B). It is presumed that the fluorescence quenching is due to the paraformaldehyde fixation step required in processing samples with the virulent strain. To visualize ClpX without paraformaldehyde fixation, we expressed *Ptet::clpX_TB_-cfp* in *M. smegmatis,* a rapidly growing and nonpathogenic mycobacterial member. Visualization of ClpX-CFP in *M. smegmatis* revealed distinct localization at the cell poles in the majority of cells and at midcell sites in a small fraction of cells (Fig. 8A). When quantified, these structures corresponded to 82.5±6.5% at the cell poles and 11±2% at midcell sites (n = 124). Next, to address whether ClpX-CFP colocalizes with FtsZ, we transformed *Pami::ftsZ-yfp* into the *M. smegmatis* strain expressing *Ptet::clpX-cfp* and visualized ClpX and FtsZ following a 1-h induction with 5 ng of anhydrotetracycline and 0.2% acetamide. As expected, FtsZ-YFP structures were evident at midcell sites and poles (Fig. 8C, see panel FtsZ-YFP), similar to our earlier published data [34]. ClpX-CFP showed similar localization (Fig. 8C, see panel ClpX-CFP). The FtsZ-YFP structures were faint and tended to bleach easily. Even with this limitation, we were able to see a colocalization pattern (see Fig. 8, panel C Merge). ClpX-FtsZ colocalization was noted at the cell poles in 12±2.8% of cells and at midcell sites in 3.5±2.1% of cells (n = 110).

## Discussion

The results presented in this study show that *M. tuberculosis* ClpX interacts with FtsZ and a possible consequence of these interactions is modulation of Z-ring assembly at midcell sites in vivo and interference with GTP-dependent FtsZ polymerization activity in vitro (Figs. 7, 8 and 2). The critical concentration of FtsZ polymerization is increased in the presence of ClpX (Fig. 2B). The intracellular levels of FtsZ are, however, unaffected upon ClpX overproduction. These results, combined with the observation that the intracellular ratio of ClpX to FtsZ is 1∶2, are consistent with a notion that in *M. tuberculosis* ClpX interacts with FtsZ at stoichiometric levels. Such an interaction could lead to sequestration of the available pools of FtsZ required for catalyzing Z-ring assembly and cell division in *M. tuberculosis*.

FtsZ localization at midcell sites and cell poles and the potential regulators affecting Z-ring assembly have been extensively studied in *E. coli*, *B. subtilis* and a handful of other bacteria (reviewed in [7]). In contrast to the situation in other bacteria, only a few proteins affecting Z-ring assembly in *M. tuberculosis* have been identified. One of these is ChiZ (Rv2719c), a cell wall hydrolase and a negative regulator of cell division [5]. The ChiZ protein has been shown to modulate Z-ring assembly by localizing its activity to midcell sites and cell poles, the same locations where FtsZ is found [5]). The ChiZ protein, however, does not interact with FtsZ; hence, the interference effects are rather indirect. The second protein is FtsW, a bona fide FtsZ-interacting partner [38] hypothesized to function as a positive regulator of Z-ring assembly [34], [38]. Genetic studies indicate that FtsW is required for productive FtsZ ring formation and that FtsW possibly functions to promote and stabilize Z-rings in mycobacteria [34]. More recently, FipA, a FtsZ interacting protein has been shown to be required for sustenance of cell division in *M. tuberculosis* under oxidative stress conditions [43]. The results presented in this study attest that *M. tuberculosis* ClpX is a bona fide interaction partner of FtsZ and potentially acts as a negative regulator of Z-ring assembly and cell division. Thus, ClpX joins the list of proteins that regulate Z-ring assembly and cell division in the human pathogen *M. tuberculosis*.

As reviewed, the modes of action of ClpX or ClpXP in cell division control are distinctly different in different bacterial species. *C. crescentus* ClpXP indirectly regulates cell division by degrading the CtrA regulator thereby modulating the expression of cell division genes [11], [44], [45]. *B. subtilis* ClpX inhibits FtsZ polymerization activity in vitro and Z-ring assembly in vivo, independent of the ClpXP protease activity [24], [25]. The physical interaction between the ClpX and FtsZ proteins in *B. subtilis*, however, was not investigated in these studies. ClpX is an abundant protein in *B. subtilis*, with 8,400 molecules per cell [46]. Thus, it is likely that ClpX operates at stoichiometric levels in affecting FtsZ activities in *B. subtilis*. While this work was in progress, two recent reports indicated contrasting roles for *E. coli* ClpX in cell division. One study concluded that ClpXP operates catalytically to degrade FtsZ polymers and monomers, thereby modulates the equilibrium between free and polymeric FtsZ required for Z-ring assembly in an ATP-dependent manner [19]. The other study reported that *E. coli* ClpX directly modulates FtsZ polymer dynamics by disassembling FtsZ polymers in an ATP-independent manner [20] and that ClpXP protease activity contributes to a minor role in cell division regulation. Therefore, our studies showing a physical interaction between ClpX and FtsZ (Figs. 3–5), the similar intracellular FtsZ levels in cells producing altered levels of ClpX (supplementary Fig. S7), the modulation of FtsZ polymerization in vitro and Z-ring assembly in vivo (Figs. 2 and 7), combined with the finding that the intracellular ratio of ClpX to FtsZ is 1∶2 (Fig. 6A), are consistent with the notion that in *M. tuberculosis* ClpX interacts with FtsZ and possibly interferes with Z-ring assembly by sequestering the available intracellular pools of FtsZ. While this reasoning assumes that overproduction of ClpX in vivo could lead to cell division arrest and extensive filamenation, in reality this might not happen (see below). For example, our results showed that a 6-fold overproduction of ClpX caused ∼50% increase in cell length. While this can be considered modest, it is noteworthy that a 2-fold increase in ClpX in *B. subtilis* resulted in only a ∼20% increase in cell length [25]. ClpX is the substrate recognition part of the ClpXP protease complex. Thus, increased levels of ClpX could promote its interaction not only with FtsZ but also with its other substrates. For example, the ClpXP complex in *E. coli* is known to associate with ∼60 proteins [23]. Therefore, while the in vitro results suggested that FtsZ and ClpX interact stoichiometrically, the extent of in vivo filamentation would likely depend on the ClpX ‘available’ for FtsZ (cell division) inhibition. The observed modulation of Z-ring assembly upon ClpX overproduction was carried out in *M. tuberculosis* background WT for *clpP1* and *clpP*2. Thus, our data also suggest that ClpX mediated modulation of FtsZ assembly is independent of the presumptive *M. tuberculosis* ClpXP protease activity as in *B. subtilis* and possibly in other bacteria.

Our studies with FtsZ mutant proteins, FtsZ_G103S_, defective in binding to GTP and, hence, defective in polymerization [28], FtsZ_D210G,_ defective in GTP hydrolysis and, hence, deficient in exhibiting polymer dynamics [47], FtsZ_D374-76A_ and FtsZ_ΔC21_ which are defective for interactions with FtsW [34], [38], and possibly other proteins, indicate that *M. tuberculosis* ClpX interacts with FtsZ independent of FtsZ oligomeric state or associations with other proteins. Parallel data showing that ClpX interferes with FtsZ polymerization activity in the presence of the non-hydrolysable GTP analog, GMPCPP [31], reduces the amount of FtsZ polymers formed even when added to preformed FtsZ polymers (Fig. S3) and the colocalization of ClpX-CFP with FtsZ-YFP at midcell Z-rings (Fig. 8) are in partial support of the above conclusions. Together, these results are consistent with a model in which *M. tuberculosis* ClpX interacts with FtsZ independent of its oligomeric state or associations with other proteins and sequesters FtsZ from engaging in its activities. This line of thinking suggests that ClpX, like SulA of *E. coli,* inhibits the FtsZ polymerization by a sequestration mechanism [31]. However, unlike ClpX, the *E. coli* SulA protein does not inhibit FtsZ polymerization in the absence of GTP hydrolysis. The *E. coli* MinC also requires GTP hydrolysis activity of FtsZ for inhibition of polymer assembly. Since MinC does not affect the GTPase activity of FtsZ it is thought that MinC requires dynamic FtsZ polymers to exert its effect [30].

Our studies raise questions as to when the consequences of FtsZ and ClpX interactions lead to modulation of Z-ring assembly in *M. tuberculosis*, especially since overproduction of ClpX led to only modest changes in cell length and growth rate (Fig. 6). To answer this question, it is important to recognize that both *M. tuberculosis* ClpX and FtsZ are relatively abundant proteins and are present at an intracellular ratio of 1∶2. ClpX is a component of the ClpXP protease and could be interacting with other hitherto unrecognized interaction partners, similar to the situation reported in *E. coli* [23]. Thus, we expect that under normal growth conditions ClpX interactions with FtsZ would not lead to interference of Z-ring assembly in *M. tuberculosis.* Limited ∼6-fold overproduction of ClpX (Fig. 6B inset), however, could impair the optimal intracellular ratio between ClpX and FtsZ. This could in turn interfere with FtsZ activities and affect Z-ring assembly. It is important to note in this regard that ClpX expression is upregulated during intracellular growth and upon exposure to antibiotic stress, the same growth conditions where the intracellular levels of FtsZ are not affected [26]. FtsZ ring assembly is modulated by these conditions, however, and cell division is delayed [26]. The expression of *clpX* is likely modulated in response to various environmental cues and the induced ClpX possibly functions as a chaperone and as part of the ClpXP protease. We propose that under some circumstances such as growth of *M. tuberculosis* in macrophages and upon exposure to antibiotic cephalexin, ClpX also modulates FtsZ-ring assembly such that the pathogen can cope with the stress of survival.

*M. tuberculosis* Z-ring assembly, as in *E. coli* and *B. subtilis* [48], [49], is a dynamic process, and the subunits in the FtsZ ring undergo constant turnover [47]. Studies have also indicated that ∼30% of the FtsZ protein pool is in the Z-ring, while the remainder is likely to be present as short polymers and monomers [49]. Therefore, it is likely that in *M. tuberculosis*, ClpX-bound FtsZ monomers are incompatible for polymerization or exchange with the subunits in FtsZ polymers or protofilaments [32], [47], [50]. This, in turn, could limit subunit exchange and prevent productive Z-ring assembly. Although further studies are required to address these issues in detail, our data suggest that ClpX could be one of the factors regulating *M. tuberculosis* Z-ring assembly during intracellular growth.

We found approximately three times more colocalization of ClpX and FtsZ at the cell poles compared to midcell sites (Fig. 8). However, our data do not rule out a possibility that colocalization of ClpX and FtsZ could also be due to their respective interactions with other proteins. And while colocalization of two proteins is not a direct indication of interaction, these data combined with bacterial two-hybrid and MPFC assays suggest that increased ClpX localization at the cell poles helps to disassemble the remnants of FtsZ polymers from new cell poles and to restrict polar ring formation. It is known that the MinCDE system is critical for targeting FtsZ to midcell sites [3]. The *M. tuberculosis* genome lacks identifiable homologs of the MinCDE system [15]. Perhaps in the absence of the MinCDE system, ClpX-FtsZ interactions could restrict Z-ring assembly to midcell sites in *M. tuberculosis*.

## Materials and Methods

### Strains and bacterial growth conditions

*E. coli* strains were grown in Luria-Bertani (LB) broth or LB agar supplemented with ampicillin (Amp, 50 µg ml^−1^) or kanamycin (Km, 50 µg ml^−1^) or hygromycin (Hyg, 200 µg ml^−1^). *M. tuberculosis* strains were propagated in Middlebrook 7H9 broth supplemented with OADC (oleic acid, albumin, dextrose, catalase with sodium chloride), 0.05% tween 80 and appropriate antibiotics (Km at 25 µg ml^−1^; Hyg at 50 µg ml^−1^). When needed, strains were plated on 7H10 agar (BD biosciences) plates containing OADC and appropriate antibiotics. Growth was monitored by measuring absorbance at 600 nm and viability by determining the colony forming units on Middlebrook 7H10 agar plates without or with anhydrotetracycline. Recombinant *M. smegmatis* or *M. tuberculosis* strains with an additional copy of extrachromosomal or integrated *clpX* are referred to as merodiploid strains. Integrating and replicating plasmids were confirmed by bead-beating followed, respectively by PCR or restriction digestion of the recovered DNA as described [5], [6].

### Molecular techniques

Oligonucleotide primers used in this study are listed in Supplementary Table S1. The *clpX* coding region was cloned in the sense orientation using primers ClpX-PacI and ClpX-SwaI or antisense orientation using asClpX-PacI and asClpX-SwaI under the inducible tetracycline (tet) promoter in pLR52, a replicating *E. coli - Mycobacterium* shuttle vector (Tables S1 and S2). As needed, *cfp* was cloned in-frame downstream of the *clp*X gene using CFP-XbaI and CFP-SwaI primers (Table S1). For some experiments, *ftsZ-gfp* or *ftsZ-yfp* fusions were cloned downstream of the tet or amidase promoter in an integrating vector (Table S2). Plasmids pUAB100 and pUAB200 (kind gift from Dr. Adries Steyn, UAB) were used for creating pMR118 and pMR119 (Table S2). For overproduction and purification of the recombinant ClpX protein, the *clp*X coding region was cloned in pET-19b vector (Novagen) and the recombinant His-ClpX fusion protein was purified on Ni-NTA affinity columns. A truncated *fts*Q gene coding for the N-term 100 aa was also cloned in pET-19b vector using primers FtsQ-NdeI and FtsQN100-BamHI (see T1-S) and the recombinant protein was purified. All constructs were verified by sequencing. Recombinant plasmids were used to transform *E. coli*, *M. smegmatis* or *M. tuberculosis* as described [50]. Constructs used for Bacterial Adenylate Cyclase-based Two-Hybrid (BACTH) and Mycobacterial Protein Fragment Complementation (M-PFC) assays were produced using the primers described in supplementary Table S1. For M-PFC assay, *ftsZ* and *clpX* were cloned under the inducible tet promoter as expression from the constitutive *hsp60* promoter caused growth defects in the host strain (Table S2; [50]). Recombinant *M. tuberculosis* and *M. smegmatis* strains were confirmed by PCR of genomic DNA or restriction digestion of recovered plasmid DNA as described [50].

### Western blotting

Intracellular lysates of *M. tuberculosis* strains were prepared, the ClpX and FtsZ levels were quantitated by immunoblotting and normalized to SigA as described [26]. Cell lysates were resolved on NuPAGE polyacrylamide gels, transferred to PVDF membrane and probed with anti- FtsZ [6], anti- His or anti- *B. subtilis* ClpX antibodies (α-ClpX_BS_, kind gift from Dr. Ulf Gerth, Germany), diluted to 1∶1000, 1∶2000 and 1∶25,000, respectively. Anti-σ^70^ antibodies (Neoclone Biotechnology, Madison, WI) that recognize SigA and anti- His-tag antibodies (Genscript) were used as recommended by the manufacturer [26]. Immunoblots were processed with the ECF Western blotting kit (GE life sciences, Piscataway, NJ) and scanned on a Bio-Rad Molecular Imager. For quantitative immunoblotting, known amounts of FtsZ or ClpX were quantified by volume analysis function of the QuantityOne software and standard curves were plotted. Lysates loaded on the same gel as the standards were then quantitated using the standard curve.

### Microscopy

Wild type (WT) and recombinant *M. tuberculosis* strains were grown for various periods of time with shaking, harvested by centrifugation, washed in phosphate buffered saline, fixed in 4% paraformaldehyde (PAF), and stored at 4°C until further use. DAPI staining was done after washing off the PAF and incubating with 0.25 µg/ml DAPI for 15 min at RT. Excess stain was removed by centrifugation, cells were resuspended in PBS and viewed immediately. Brightfield and fluorescence imaging was done on a Nikon Eclipse 600 microscope equipped with a 100X Nikon Plan Fluor oil immersion objective with a numerical aperture of 1.4. The following filter sets were used for microscopy: GFP - standard fluorescein isothiocyanate filter set (*Ex*_484–499_, *Em*_459–509_, Chroma Technology); CFP (*Ex*_426–446_, *Em*_460–500_, Nikon); YFP (*Ex*_490–510_, *Em*_520–550_, Nikon); DAPI- DAPI filter set (*Ex*_325–375,_ *Em*_435–485_, Chroma Technology). All images were acquired with a Photometrics Coolsnap ES camera and Metamorph 6.2 imaging software (Universal Imaging Corporation) and optimized with Adobe Photoshop 7.0. It is noteworthy that bright focused localization was obtained in some strains but not in others. Since all strains were grown and processed under similar conditions, we tend to favor an argument that diffuse signals in particular strains are related to the experimental context and are not an artifact of microscopy.

### Purification of FtsZ and ClpX

His-tag fusion recombinant proteins FtsZ (pSAR1), FtsZ_G103S_ (pRR3) or FtsZ_D210G_ (pRR7), FtsZ_D374-76A_ (pLR12), FtsZΔC21 (pMK13) were purified under soluble conditions on Ni-NTA columns as described [28]. As needed, His-tag was removed by incubating the recombinant proteins with thrombin for 8 h at 4°C and the tag-free protein was recovered in the flow-through fractions on Ni-NTA column. The ClpX (pRD21) protein was prepared by isolation and subsequent solubilization of inclusion bodies using 8 M Urea. The resulting ClpX was then refolded by addition of the protein to a volume of refolding buffer containing 0.4 M L-arginine and 2% glycine to yield a final protein concentration of 0.5 mg/ml. The refolded ClpX was dialyzed and applied to a DEAE Sephacel column in column buffer (50 mM Tris-HCl pH 8.0, 50 mM NaCl and 10% glycerol), washed with column buffer, and eluted with the same buffer containing 1 M NaCl. Peak ClpX fractions were pooled, dialyzed and applied to a Ni-NTA column, washed, and eluted with 1X binding buffer (50 mM Sodium Phosphate pH 7.8, 500 mM NaCl, 10% Glycerol, 5 mM 2-Mercaptoethanol) containing I M Imidazole. Final preparations of ClpX protein were dialyzed against the storage buffer (25 mM HEPES-NaOH pH 7.2, 0.1 mM EDTA, 1 mM DTT, 50 mM NaCl, and 10% glycerol). This ClpX refolding protocol yielded protein concentrations in the range of 7 to 8 µM and the protein obtained was used in various experiments described in this study. Our innumerable attempts to obtain a more concentrated ClpX protein stock were, however, not successful. Size exclusion chromatography of purified ClpX on Superdex 200 10/300 GL column revealed that the majority of ClpX was hexameric (data not shown). Unless otherwise mentioned, the molar concentrations of ClpX in the various assays are based on monomer molecular weight.

### FtsZ polymerization assays

#### Light scatter assays

FtsZ polymerization was examined by the right angle light scatter assay [27]. Briefly, various concentrations of FtsZ were incubated without or with ClpX protein (molarity based on monomer concentration) in 50 mM MES buffer pH 6.5 containing 100 mM KCl, 5 mM MgCl_2_ and 1 mM ATP at 30°C. Polymerization was initiated by the addition of GTP to 1 mM. The change in light scatter was monitored in FP6500 fluorimeter at 400 nM using a 1 nM slit. The data points were collected every 5 s for 15 min and plotted using Excel. Buffer controls for polymerization assays contained ClpX storage buffer lacking ClpX protein and the scatter data obtained were used for data normalization.

#### Sedimentation assay

Polymerization reactions containing FtsZ in a final volume of 50 µl were incubated at 30°C for 10 min and the polymerized FtsZ was collected by centrifugation at 80K for 10 min at 4°C. As needed, various concentrations of ClpX were added to the reaction. Supernatant and pellet fractions were separated on sodium dodecyl sulfate polyacrylamide (SDS-PA) gels, stained with Coomassie blue and scanned in a BioRad Molecular Imager using the QuantityOne software. Standard curve prepared with pure FtsZ protein was used for quantitating the FtsZ in the pellet fractions of the sedimentation assay. In some sedimentation assays 3′-(N-Methyl-anthraniloyl)-2′-deoxy-guanosine-5′-triphosphate, GMPCPP (Jena Biosciences, Germany), was used at a final concentration of 1 mM [30], [31]. As for light scatter assays, controls reactions contained an equivalent volume of ClpX storage buffer instead of the ClpX protein.

### Protein-protein interaction assays

#### Pull-down assays: Experiments with pure proteins

Equimolar amounts of tag-free FtsZ and His-ClpX were mixed and incubated for 20 min, applied onto Ni-NTA resin in 1X binding buffer (50 mM Sodium Phosphate pH 7.8, 10% Glycerol, 5 mM 2-Mercaptoethanol, 150 mM NaCl, 0.1% CHAPS (3-[(3- Cholamidopropyl)dimethylammonio]-1- propanesulfonate) and 1% Nonidet P-40), washed 7X with 400 µl of 1X binding buffer containing 20 mM imidazole and eluted with 0.3 M imidazole. For assessing the strength of FtsZ-ClpX interactions, pull-down assays were performed in the presence of 0.2 or 0.5 M NaCl. Samples run on duplicate gels were probed with either α-FtsZ or α-ClpX_BS_ antibodies as described above. We noted that the eluted FtsZ is not stoichimetric to His-ClpX, presumably due to loss during extensive washing steps.

#### Experiments with cell lysates

*E. coli* strains expressing full length His-ClpX (pRD21) together with S-tag-FtsZ (pLR66; Table S2) under the T7 promoter were induced, clear cellular lysates were prepared in 1X binding buffer and applied to Ni-NTA resin. Bound proteins were eluted and loaded on NuPAGE gels and analyzed by immunoblotting as described above.

#### Solid Phase assays

Four micrograms of purified ClpX or BSA was allowed to adsorb overnight onto the wells of a microtiter plate. After washing the excess unbound protein with phosphate-buffered saline containing 0.5% Tween-20 (PBST), the wells were blocked with 1% BSA in PBST for 1 hr at room temperature and incubated for 2 hrs with varying concentrations of FtsZ protein. Unbound FtsZ was removed and wells washed 5X with PBST. FtsZ bound to ClpX was detected with anti-FtsZ_TB_ antibodies by ELISA (AnaSpec). Wells with BSA served as negative controls.

#### Mycobacterial protein fragment complementation (M-PFC) assay

Recombinant *M. smegmatis* expressing *ftsZ-dhfr[F1,2]/ftsZ-dhfr[F3]; clpX-dhfr [1], [2]/ftsZ-dhfr[3]; gcn4-dhfr[1], [2]/gcn4-dhfr[3]; clpX-dhfr[1], [2]/gcn4-dhfr[3]; dhfr[1], [2]/dhfr[3] or clpXΔN200-dhfr[1], [2]/ftsZ-dhfr[3]* were selected on 7H11 agar plates containing Hyg and Km. Single colonies were patched on 7H11 plates with appropriate antibiotics in the presence/absence of various concentrations of Trimethoprim (Trim). Growth on Trim plates indicated interaction. Positive interactions were further confirmed by alamar blue assay [37]. Briefly, *M. smegmatis* strains expressing interacting proteins were cultured in 0.1 ml of 7H9 broth in 96-well, untreated, white polystyrene Nunc plates. All strains except *ftsZ-dhfr [F1,2]/ftsZ-dhfr[F3]* were grown with 10 ng/ml anhydrotetracycline (tet) for 3 h. Various concentrations of Trim were added for an additional 24 hrs at 37°C and finally incubated with 5 µl of alamar blue for 6 hrs. The plates were read in a Varian Cary Eclipse Fluorescence Spectrophotometer (*Ex_530_ and Em_590_*). *M. smegmatis* with *gcn4-dhfr[1], [2]/gcn4-dhfr[3]* served as control.

#### Bacterial two hybrid (BACTH) assay

BACTH system [35] kit was purchased from Euromedex and used as recommended. *E. coli* BTH101 recombinants with various combinations of plasmids (see Table S2) were selected on MacConkey agar supplemented with 100 µg/ml Amp and 50 µg/ml Km at 30°C for 24 to 36 h. For evaluating the strength of interaction, β-galactosidase activity was measured with cells grown in LB broth and samples were processed as recommended by the supplier. Reactions were started by the addition of 0.25 ml of 0.4% o-nitrophenol-β-galactoside (ONPG) and the tubes incubated at 28°C for 5 min or until a visible yellow color developed. Reactions were stopped by the addition of 0.5 ml of 1 M Na_2_CO_3_ and OD_420_ was recorded [35]. The enzymatic activity was defined as units per milliliter: 200× [(OD_420_ of the culture - OD_420_ in the control tube)/minutes of incubation] × dilution factor. The specific activity of β-galactosidase is defined as units/mg dry weight bacteria and 1 unit corresponds to 1 nmol of ONPG hydrolyzed per min at 28°C. At least 5-fold higher β-galactosidase activity than that measured for BTH101 carrying a single gene and an empty vector was considered indicative of an interaction. *E. coli* BTH101 transformants obtained with pKT25-zip and pUT18C-zip served as positive controls for complementation (Table S2).

### RNA extraction and RT-qPCR

RNA was prepared from exponential cultures of *M. tuberculosis* strains grown in vitro or in macrophages essentially as described [51]. For some experiments RNA was isolated from WT *M. tuberculosis* exposed to 20 mg ml^−1^ cephalexin and 30 mg ml^−1^ lithium clavulanate for 24 hrs. For extraction of RNA from intracellular bacteria, macrophage monolayers were separated 3 days after infection, washed, suspended directly in RNAzol and processed as described [5], [51], [52]. Extraction of total RNA and synthesis of complementary strand DNA from mRNA specific to 16S rRNA and *clp*X using reverse transcription primers and iScript kit (BioRad) were as described [5], [51], [52]. Quantitative real time PCR (TaqMan chemistry) was carried out in a BioRad ICycler using the Taq DNA polymerase (NEB). The calculated threshold cycle (Ct) value for *clp*X was normalized to the Ct value for 16S rRNA and the fold expression was calculated using the formula: Fold change  = 2^Δ(ΔCt)^ [51]. No RT RNA samples were included as negative controls. Expression data are average from 3 independent RNA preparations, each reverse transcribed and quantitated by real time PCR in triplicate. Real-time PCR conditions: initial activation at 95°C for 3 minutes; followed by 40 cycles of 95°C for 10 sec and 55°C for 30 seconds.

## Supporting Information

Figure S1 FtsZ assembly is not affected by RecA: Light scatter assay for FtsZ polymerization in the presence or absence of RecA. Reactions containing 7.5 µM FtsZ mixed with storage buffer alone or storage buffer containing 3 µM RecA were initiated with GTP to a final concentration of 1 mM and followed for 15 minutes. Neither the rate and nor the extent of FtsZ polymerization was affected by RecA.
(0.17 MB TIF)

Figure S2 ClpX inhibition of FtsZ assembly: FtsZ polymerization was examined by the sedimentation assay. Reactions contained varying amounts of FtsZ polymerized without (A) or with a fixed concentration of ClpX at 2 µM (B). Polymerized FtsZ was collected by centrifugation and supernatant (S) and pellet (P) fractions were loaded on SDS-PA and visualized by Coomassie staining.
(0.15 MB TIF)

Figure S3 Addition of ClpX interferes to the preformed FtsZ polymers: Sedimentation assay was performed as described above with 5.4 µM FtsZ and 2 µM ClpX. For reactions 2 and 3, ClpX or storage buffer, respectively, was added at the same time as FtsZ. For reactions 4 and 5, FtsZ polymers were preformed for 5 minutes and then ClpX or storage buffer was added. Some fraction of ClpX in the pellet fractions is presumably due to its oligomerization/aggregation [20].
(0.21 MB TIF)

Figure S4 Characterization of ClpX-FtsZ interaction: (A) Strength of FtsZ-ClpX interaction. Pull-down assay using His-ClpX and tag-free FtsZ was performed in buffers containing 0.2 M and 0.5 M NaCl (see materials and methods for details). Load (L), wash (W) and elution (E) fractions were analyzed by immunoblotting. (B) FtsZ-ClpX complex isolated from cell free lysates. Cellular lysates from *E. coli* strain expressing *his-clpX_TB_* and *ftsZ_TB_-S-tag* were loaded on NiNTA column and pull-down assay performed as described in the text. Load (L), wash (W) and elution (W) fractions were analyzed by immunoblotting as described above.
(1.41 MB TIF)

Figure S5 ClpXΔN200 does not interact with FtsZ in BACTH (A) and MPFC (B) assays. ClpXΔN200 was cloned in BACTH or MPFC vectors (Table 1) and used along with respective FtsZ constructs in the two hybrid assays as described for figures 4A and 4B.
(0.24 MB TIF)

Figure S6 *ClpX* expression in *M.tuberculosis* antisense ClpX strain. Antisense ClpX strain was propagated in Middlebrook 7H9 broth, RNA was extracted and *clpX* mRNA levels were determined by quantitative real time PCR. *clpX* mRNA levels were normalized to 16S rRNA and data are expressed with respect to levels in WT strain grown in broth. Mean ± SD from two independent experiments are shown.
(0.14 MB TIF)

Figure S7 Overproduction of ClpX does not affect intracellular FtsZ levels. FtsZ levels in *M. tuberculosis clpX* overexpression (A) and underexpression (B) strains were examined by quantitative immunoblotting. *M. tuberculosis* cellular lysates were resolved on a 10% NuPage gel, transferred to PVDF membrane and probed with α-FtsZ or α-sigma70 antibodies. FtsZ and SigA bands were quantitated using the volume analysis function of the QuantityOne Software.
(0.24 MB TIF)

Figure S8 The viability of *M. tuberculosis* merodiploids overproducing ClpX (ClpX) and depleted in ClpX (asClpX). Broth-grown strains were spread on the plates containing 100 ng/ml anhydrotetracycline. Grown colonies were counted and data plotted using Microsoft Excel. Mean ± SD from three independent experiments are shown.
(0.13 MB TIF)

Table S1 (0.07 MB DOC)

Table S2 (0.11 MB DOC)

## References

[1] Gandhi NR, Moll A, Sturm AW, Pawinski R, Govender T (2006). Extensively drug-resistant tuberculosis as a cause of death in patients co-infected with tuberculosis and HIV in a rural area of South Africa.. Lancet.

[2] Smith I (2003). Mycobacterium tuberculosis pathogenesis and molecular determinants of virulence.. Clin Microbiol Rev.

[3] Margolin W (2000). Themes and variations in prokaryotic cell division.. FEMS Microbiol Rev.

[4] Romberg L, Levin PA (2003). Assembly dynamics of the bacterial cell division protein FTSZ: poised at the edge of stability.. Annu Rev Microbiol.

[5] Chauhan A, Lofton H, Maloney E, Moore J, Fol M (2006). Interference of Mycobacterium tuberculosis cell division by Rv2719c, a cell wall hydrolase.. Mol Microbiol.

[6] Dziadek J, Rutherford SA, Madiraju MV, Atkinson MA, Rajagopalan M (2003). Conditional expression of Mycobacterium smegmatis ftsZ, an essential cell division gene.. Microbiology.

[7] Errington J, Daniel RA, Scheffers DJ (2003). Cytokinesis in bacteria.. Microbiol Mol Biol Rev.

[8] Del Sol R, Mullins JG, Grantcharova N, Flardh K, Dyson P (2006). Influence of CrgA on assembly of the cell division protein FtsZ during development of Streptomyces coelicolor.. J Bacteriol.

[9] Frees D, Savijoki K, Varmanen P, Ingmer H (2007). Clp ATPases and ClpP proteolytic complexes regulate vital biological processes in low GC, Gram-positive bacteria.. Mol Microbiol.

[10] Gottesman S (2003). Proteolysis in bacterial regulatory circuits.. Annu Rev Cell Dev Biol.

[11] Jenal U, Fuchs T (1998). An essential protease involved in bacterial cell-cycle control.. EMBO J.

[12] Hanson PI, Whiteheart SW (2005). AAA+ proteins: have engine, will work.. Nat Rev Mol Cell Biol.

[13] Kannan N, Haste N, Taylor SS, Neuwald AF (2007). The hallmark of AGC kinase functional divergence is its C-terminal tail, a cis-acting regulatory module.. Proc Natl Acad Sci U S A.

[14] Singh SK, Rozycki J, Ortega J, Ishikawa T, Lo J (2001). Functional domains of the ClpA and ClpX molecular chaperones identified by limited proteolysis and deletion analysis.. J Biol Chem.

[15] Cole ST, Brosch R, Parkhill J, Garnier T, Churcher C (1998). Deciphering the biology of Mycobacterium tuberculosis from the complete genome sequence.. Nature.

[16] Engels S, Schweitzer JE, Ludwig C, Bott M, Schaffer S (2004). clpC and clpP1P2 gene expression in Corynebacterium glutamicum is controlled by a regulatory network involving the transcriptional regulators ClgR and HspR as well as the ECF sigma factor sigmaH.. Mol Microbiol.

[17] Viala J, Mazodier P (2002). ClpP-dependent degradation of PopR allows tightly regulated expression of the clpP3 clpP4 operon in Streptomyces lividans.. Mol Microbiol.

[18] Chien P, Perchuk BS, Laub MT, Sauer RT, Baker TA (2007). Direct and adaptor-mediated substrate recognition by an essential AAA+ protease.. Proc Natl Acad Sci U S A.

[19] Camberg JL, Hoskins JR, Wickner S (2009). ClpXP protease degrades the cytoskeletal protein, FtsZ, and modulates FtsZ polymer dynamics.. Proc Natl Acad Sci U S A.

[20] Sugimoto S, Yamanaka K, Nishikori S, Miyagi A, Ando T (2010). AAA+ chaperone ClpX regulates dynamics of prokaryotic cytoskeletal protein FtsZ.. J Biol Chem.

[21] Farrell CM, Grossman AD, Sauer RT (2005). Cytoplasmic degradation of ssrA-tagged proteins.. Mol Microbiol.

[22] Lu C, Stricker J, Erickson HP (1998). FtsZ from Escherichia coli, Azotobacter vinelandii, and Thermotoga maritima—quantitation, GTP hydrolysis, and assembly.. Cell Motil Cytoskeleton.

[23] Flynn JM, Neher SB, Kim YI, Sauer RT, Baker TA (2003). Proteomic discovery of cellular substrates of the ClpXP protease reveals five classes of ClpX-recognition signals.. Mol Cell.

[24] Haeusser DP, Lee AH, Weart RB, Levin PA (2009). ClpX inhibits FtsZ assembly in a manner that does not require its ATP hydrolysis-dependent chaperone activity.. J Bacteriol.

[25] Weart RB, Nakano S, Lane BE, Zuber P, Levin PA (2005). The ClpX chaperone modulates assembly of the tubulin-like protein FtsZ.. Mol Microbiol.

[26] Chauhan A, Madiraju MV, Fol M, Lofton H, Maloney E (2006). Mycobacterium tuberculosis cells growing in macrophages are filamentous and deficient in FtsZ rings.. J Bacteriol.

[27] Mukherjee A, Lutkenhaus J (1999). Analysis of FtsZ assembly by light scattering and determination of the role of divalent metal cations.. J Bacteriol.

[28] Rajagopalan M, Atkinson MA, Lofton H, Chauhan A, Madiraju MV (2005). Mutations in the GTP-binding and synergy loop domains of Mycobacterium tuberculosis ftsZ compromise its function in vitro and in vivo.. Biochem Biophys Res Commun.

[29] Galletto R, Kowalczykowski SC (2007). RecA.. Curr Biol.

[30] Dajkovic A, Lan G, Sun SX, Wirtz D, Lutkenhaus J (2008). MinC spatially controls bacterial cytokinesis by antagonizing the scaffolding function of FtsZ.. Curr Biol.

[31] Dajkovic A, Mukherjee A, Lutkenhaus J (2008). Investigation of regulation of FtsZ assembly by SulA and development of a model for FtsZ polymerization.. J Bacteriol.

[32] White EL, Ross LJ, Reynolds RC, Seitz LE, Moore GD (2000). Slow polymerization of Mycobacterium tuberculosis FtsZ.. J Bacteriol.

[33] Hett EC, Chao MC, Deng LL, Rubin EJ (2008). A mycobacterial enzyme essential for cell division synergizes with resuscitation-promoting factor.. PLoS Pathog.

[34] Rajagopalan M, Maloney E, Dziadek J, Poplawska M, Lofton H (2005). Genetic evidence that mycobacterial FtsZ and FtsW proteins interact, and colocalize to the division site in Mycobacterium smegmatis.. FEMS Microbiol Lett.

[35] Karimova G, Dautin N, Ladant D (2005). Interaction network among Escherichia coli membrane proteins involved in cell division as revealed by bacterial two-hybrid analysis.. J Bacteriol.

[36] Datta P, Dasgupta A, Singh AK, Mukherjee P, Kundu M (2006). Interaction between FtsW and penicillin-binding protein 3 (PBP3) directs PBP3 to mid-cell, controls cell septation and mediates the formation of a trimeric complex involving FtsZ, FtsW and PBP3 in mycobacteria.. Mol Microbiol.

[37] Singh A, Mai D, Kumar A, Steyn AJ (2006). Dissecting virulence pathways of Mycobacterium tuberculosis through protein-protein association.. Proc Natl Acad Sci U S A.

[38] Datta P, Dasgupta A, Bhakta S, Basu J (2002). Interaction between FtsZ and FtsW of Mycobacterium tuberculosis.. J Biol Chem.

[39] Ma X, Margolin W (1999). Genetic and functional analyses of the conserved C-terminal core domain of Escherichia coli FtsZ.. J Bacteriol.

[40] Mosyak L, Zhang Y, Glasfeld E, Haney S, Stahl M (2000). The bacterial cell-division protein ZipA and its interaction with an FtsZ fragment revealed by X-ray crystallography.. EMBO J.

[41] Corbin BD, Wang Y, Beuria TK, Margolin W (2007). Interaction between cell division proteins FtsE and FtsZ.. J Bacteriol.

[42] Sassetti CM, Boyd DH, Rubin EJ (2003). Genes required for mycobacterial growth defined by high density mutagenesis.. Mol Microbiol.

[43] Sureka K, Hossain T, Mukherjee P, Chatterjee P, Datta P (2010). Novel role of phosphorylation-dependent interaction between FtsZ and FipA in mycobacterial cell division.. PLoS One.

[44] Goley ED, Iniesta AA, Shapiro L (2007). Cell cycle regulation in Caulobacter: location, location, location.. J Cell Sci.

[45] Kelly AJ, Sackett MJ, Din N, Quardokus E, Brun YV (1998). Cell cycle-dependent transcriptional and proteolytic regulation of FtsZ in Caulobacter.. Genes Dev.

[46] Gerth U, Kirstein J, Mostertz J, Waldminghaus T, Miethke M (2004). Fine-tuning in regulation of Clp protein content in Bacillus subtilis.. J Bacteriol.

[47] Chen Y, Anderson DE, Rajagopalan M, Erickson HP (2007). Assembly dynamics of Mycobacterium tuberculosis FtsZ.. J Biol Chem.

[48] Anderson DE, Gueiros-Filho FJ, Erickson HP (2004). Assembly dynamics of FtsZ rings in Bacillus subtilis and Escherichia coli and effects of FtsZ-regulating proteins.. J Bacteriol.

[49] Stricker J, Maddox P, Salmon ED, Erickson HP (2002). Rapid assembly dynamics of the Escherichia coli FtsZ-ring demonstrated by fluorescence recovery after photobleaching.. Proc Natl Acad Sci U S A.

[50] Dziadek J, Madiraju MV, Rutherford SA, Atkinson MA, Rajagopalan M (2002). Physiological consequences associated with overproduction of Mycobacterium tuberculosis FtsZ in mycobacterial hosts.. Microbiology.

[51] Fol M, Chauhan A, Nair NK, Maloney E, Moomey M (2006). Modulation of Mycobacterium tuberculosis proliferation by MtrA, an essential two-component response regulator.. Mol Microbiol.

[52] Nair N, Dziedzic R, Greendyke R, Muniruzzaman S, Rajagopalan M (2009). Synchronous replication initiation in novel Mycobacterium tuberculosis dnaA cold-sensitive mutants.. Mol Microbiol.

