# Supplementary figures and images for: Mycobacterium tuberculosis ClpX Interacts with FtsZ and Interferes with FtsZ Assembly
Source: PLoS One. 2010 Jul 6;5(7):e11058. doi: 10.1371/journal.pone.0011058 (PMC2897852; doi:10.1371/journal.pone.0011058)

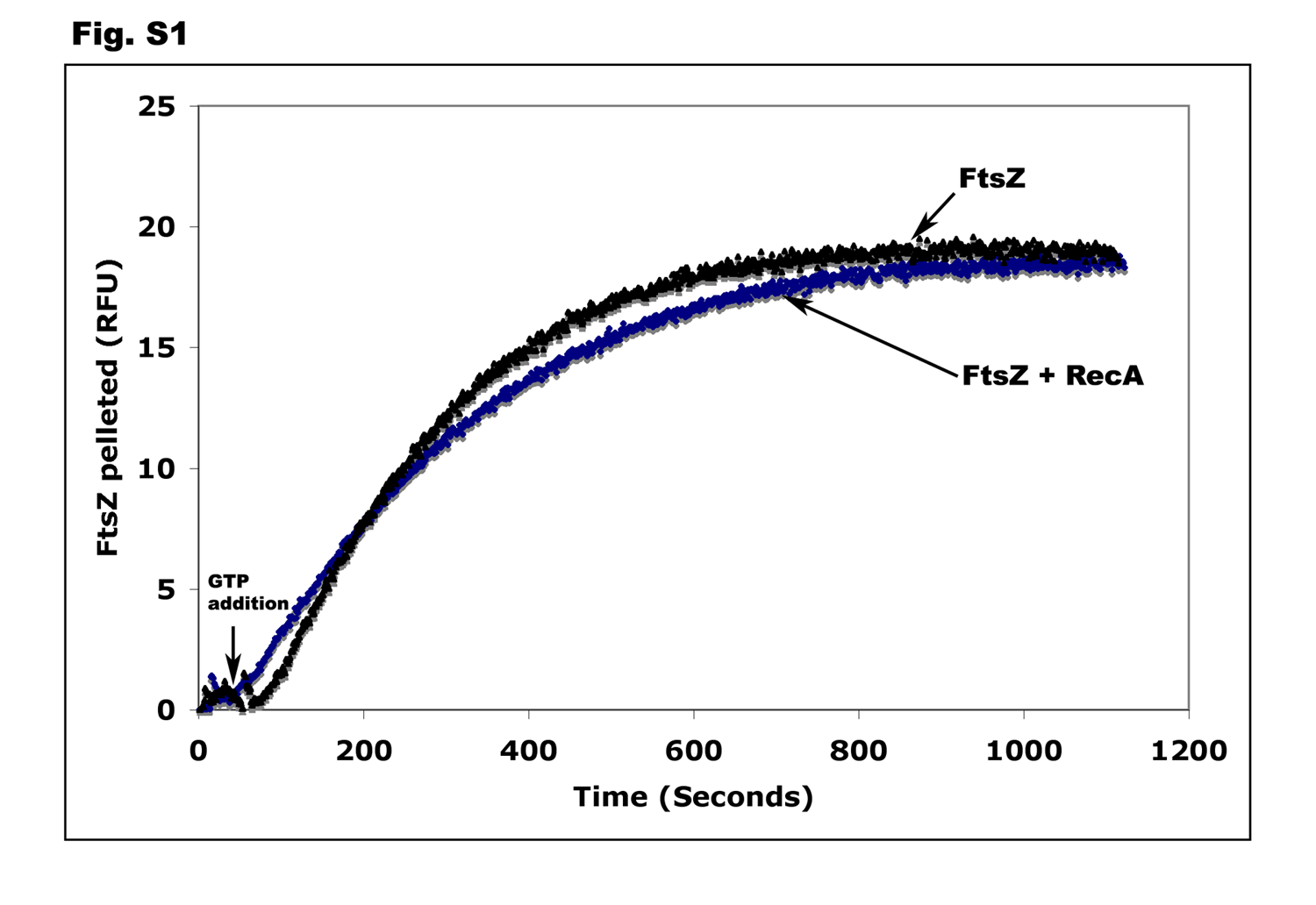

Supplement: Figure S1 — FtsZ assembly is not affected by RecA: Light scatter assay for FtsZ polymerization in the presence or absence of RecA. Reactions containing 7.5 µM FtsZ mixed with storage buffer alone or storage buffer containing 3 µM RecA were initiated with GTP to a final concentration of 1 mM and followed for 15 minutes. Neither the rate and nor the extent of FtsZ polymerization was affected by RecA. (0.17 MB TIF) [file pone.0011058.s001.tif]

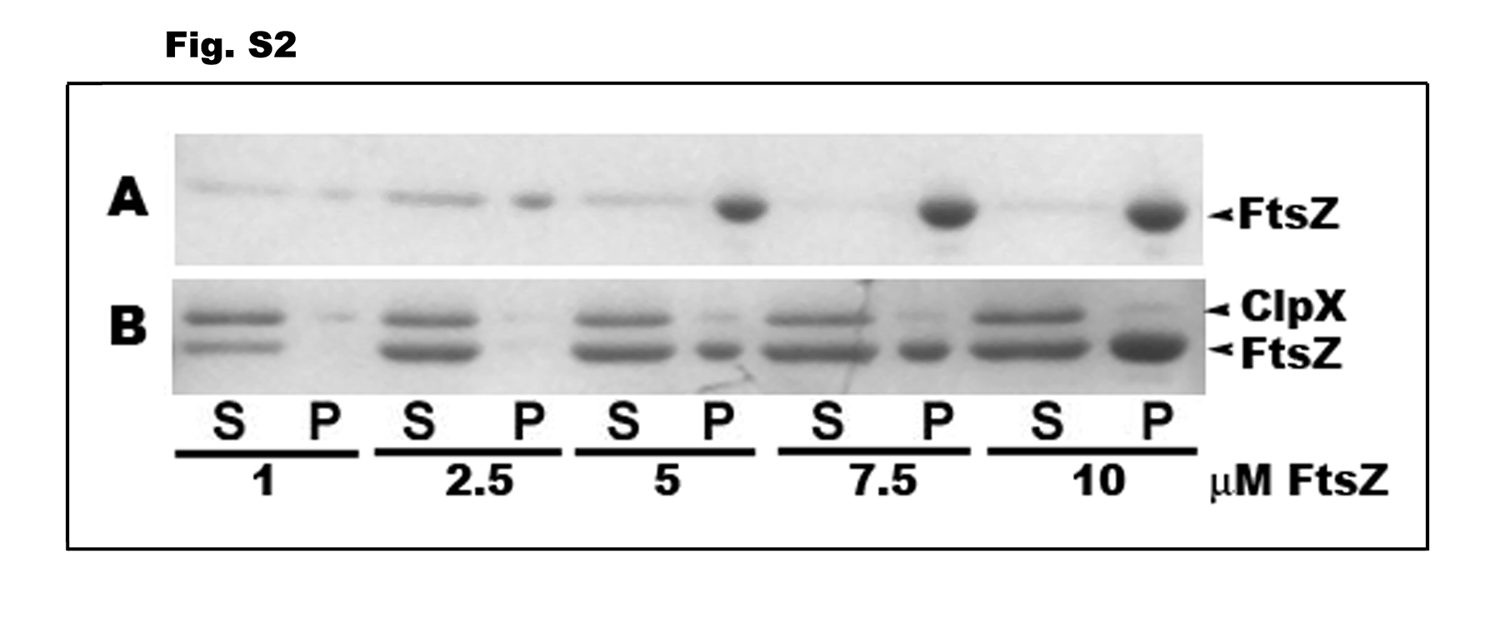

Supplement: Figure S2 — ClpX inhibition of FtsZ assembly: FtsZ polymerization was examined by the sedimentation assay. Reactions contained varying amounts of FtsZ polymerized without (A) or with a fixed concentration of ClpX at 2 µM (B). Polymerized FtsZ was collected by centrifugation and supernatant (S) and pellet (P) fractions were loaded on SDS-PA and visualized by Coomassie staining. (0.15 MB TIF) [file pone.0011058.s002.tif]

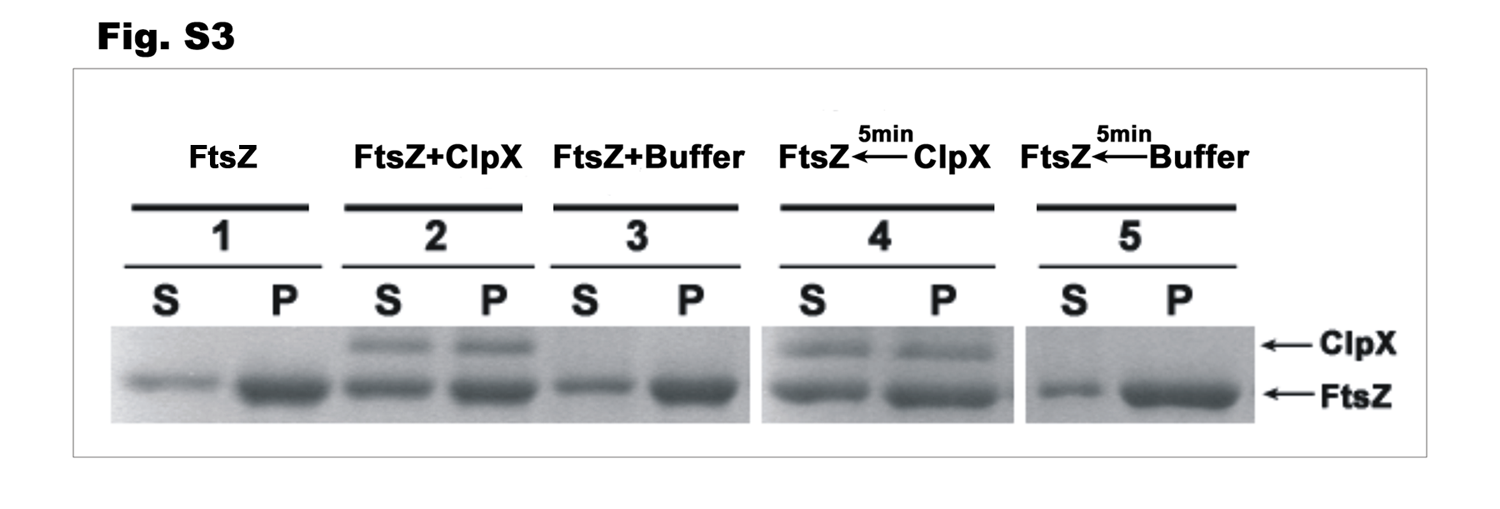

Supplement: Figure S3 — Addition of ClpX interferes to the preformed FtsZ polymers: Sedimentation assay was performed as described above with 5.4 µM FtsZ and 2 µM ClpX. For reactions 2 and 3, ClpX or storage buffer, respectively, was added at the same time as FtsZ. For reactions 4 and 5, FtsZ polymers were preformed for 5 minutes and then ClpX or storage buffer was added. Some fraction of ClpX in the pellet fractions is presumably due to its oligomerization/aggregation [20]. (0.21 MB TIF) [file pone.0011058.s003.tif]

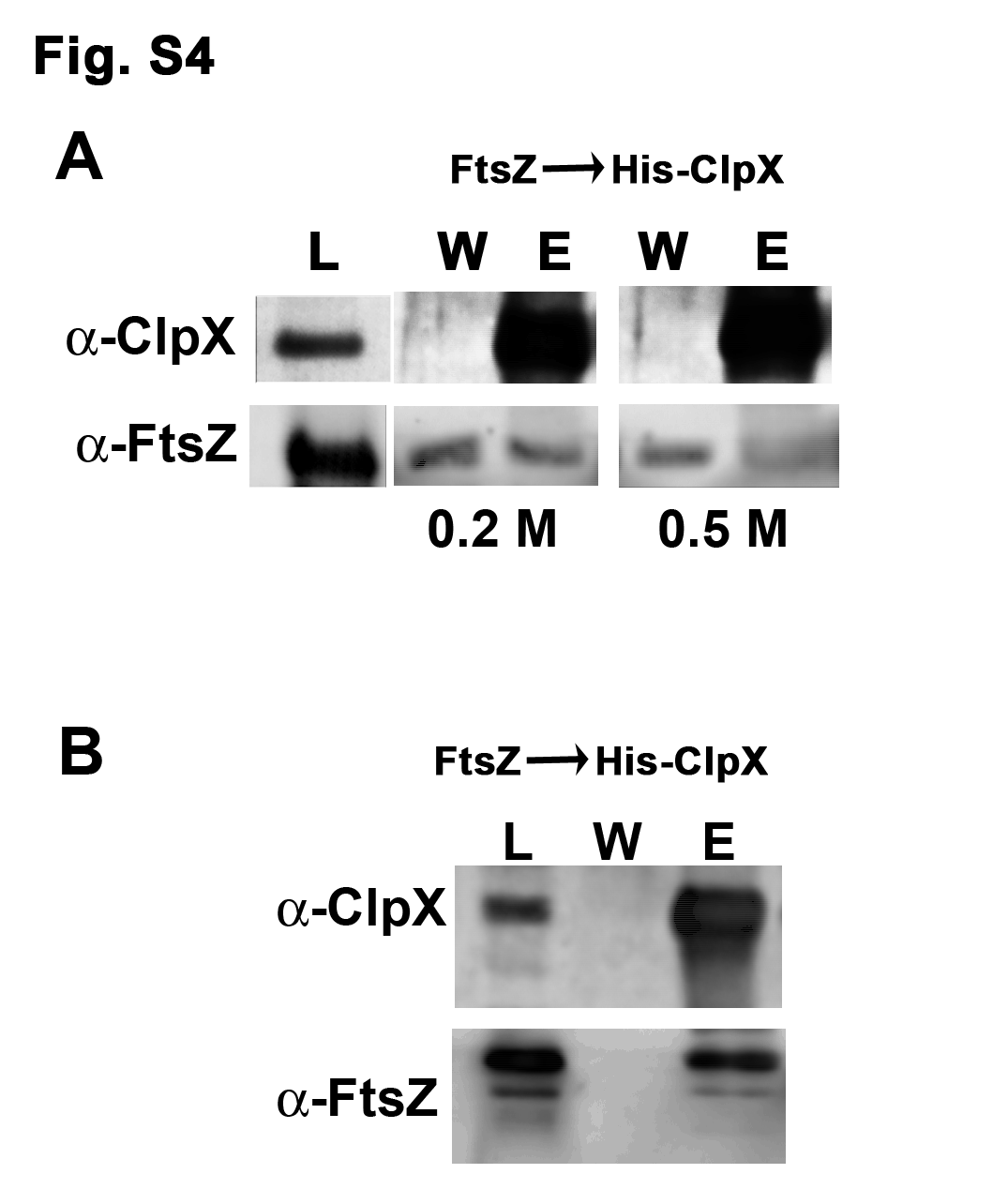

Supplement: Figure S4 — Characterization of ClpX-FtsZ interaction: (A) Strength of FtsZ-ClpX interaction. Pull-down assay using His-ClpX and tag-free FtsZ was performed in buffers containing 0.2 M and 0.5 M NaCl (see materials and methods for details). Load (L), wash (W) and elution (E) fractions were analyzed by immunoblotting. (B) FtsZ-ClpX complex isolated from cell free lysates. Cellular lysates from E. coli strain expressing his-clpXTB and ftsZTB-S-tag were loaded on NiNTA column and pull-down assay performed as described in the text. Load (L), wash (W) and elution (W) fractions were analyzed by immunoblotting as described above. (1.41 MB TIF) [file pone.0011058.s004.tif]

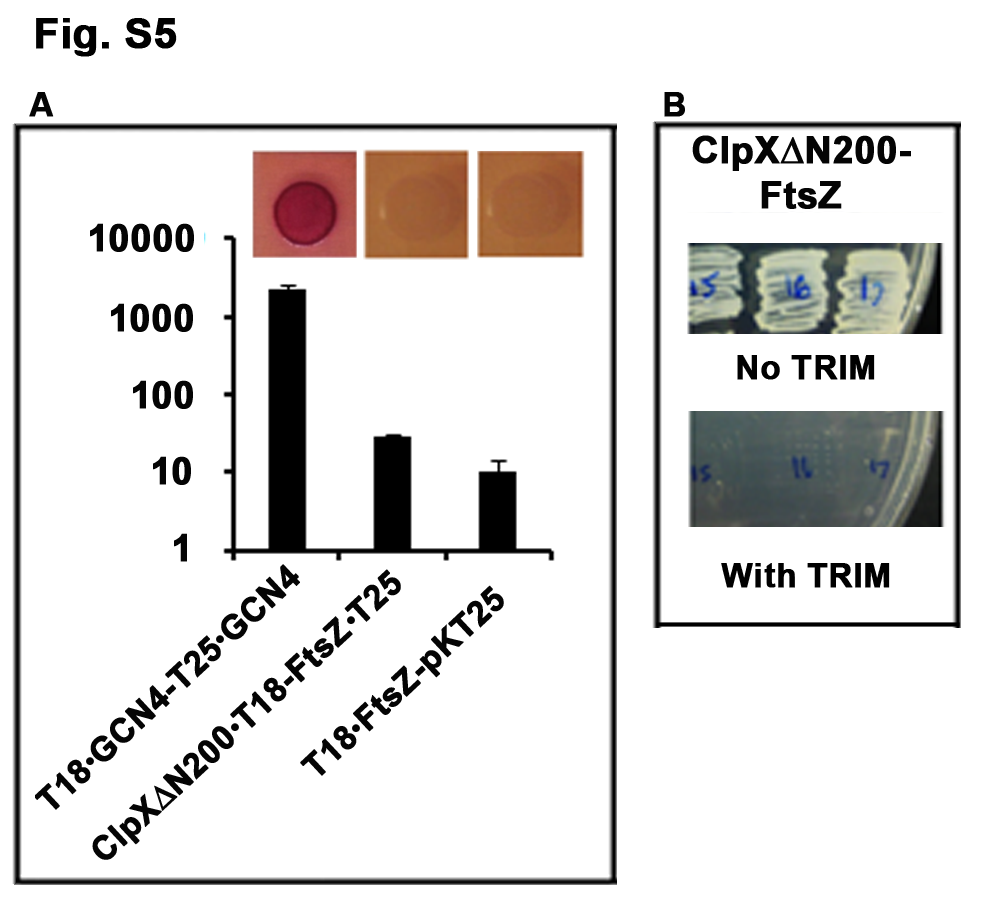

Supplement: Figure S5 — ClpXΔN200 does not interact with FtsZ in BACTH (A) and MPFC (B) assays. ClpXΔN200 was cloned in BACTH or MPFC vectors (Table 1) and used along with respective FtsZ constructs in the two hybrid assays as described for figures 4A and 4B. (0.24 MB TIF) [file pone.0011058.s005.tif]

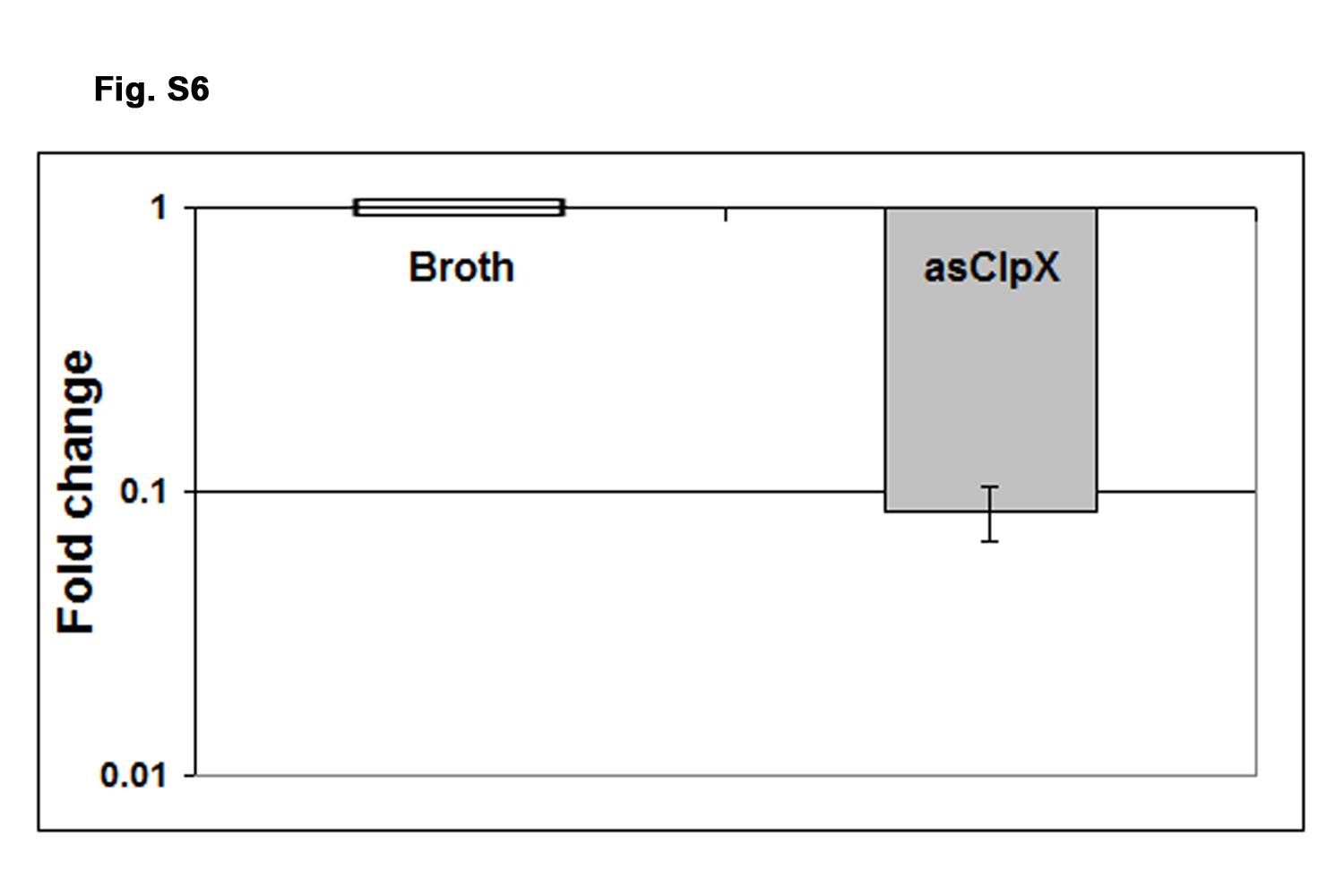

Supplement: Figure S6 — ClpX expression in M.tuberculosis antisense ClpX strain. Antisense ClpX strain was propagated in Middlebrook 7H9 broth, RNA was extracted and clpX mRNA levels were determined by quantitative real time PCR. clpX mRNA levels were normalized to 16S rRNA and data are expressed with respect to levels in WT strain grown in broth. Mean ± SD from two independent experiments are shown. (0.14 MB TIF) [file pone.0011058.s006.tif]

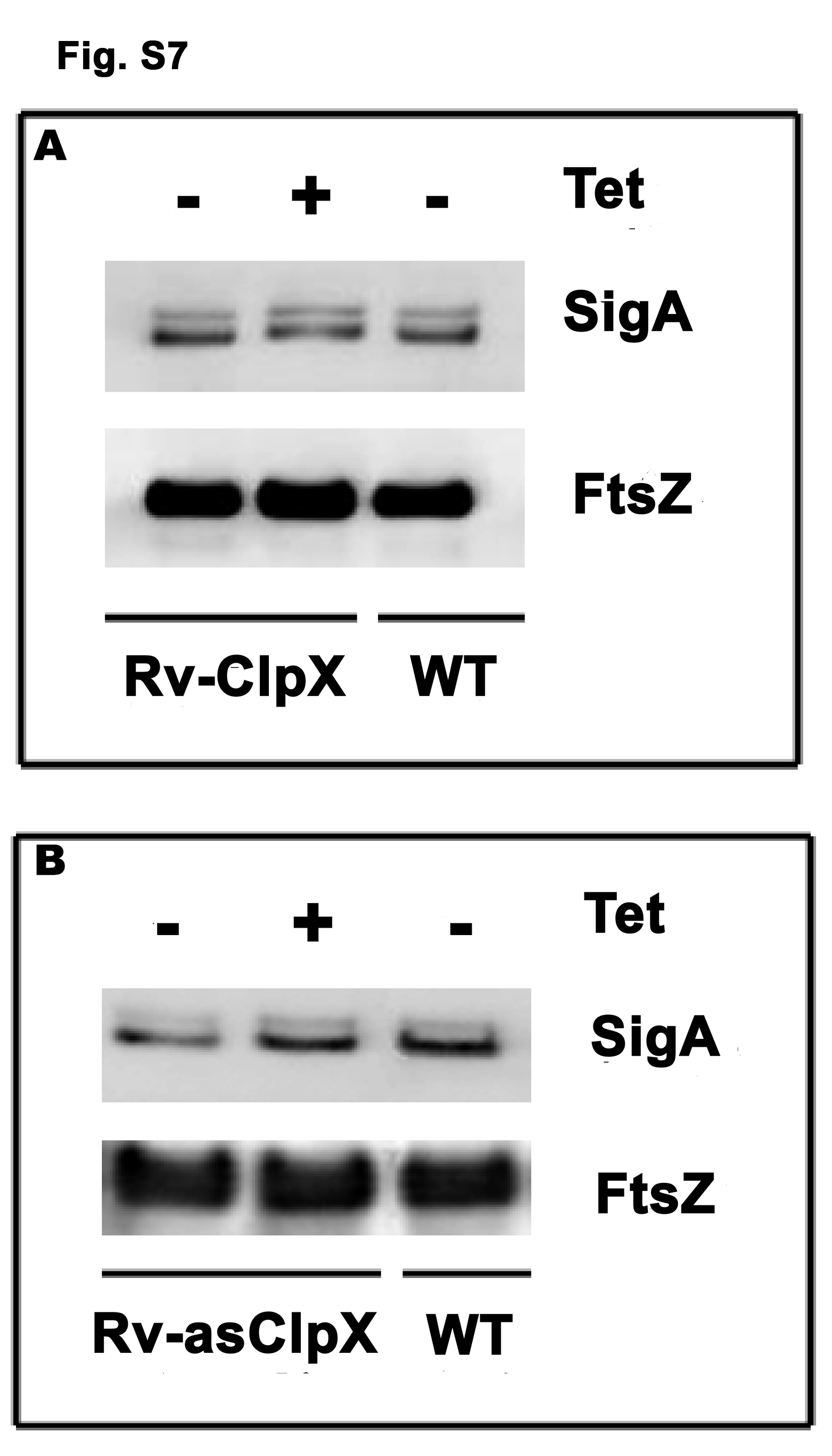

Supplement: Figure S7 — Overproduction of ClpX does not affect intracellular FtsZ levels. FtsZ levels in M. tuberculosis clpX overexpression (A) and underexpression (B) strains were examined by quantitative immunoblotting. M. tuberculosis cellular lysates were resolved on a 10% NuPage gel, transferred to PVDF membrane and probed with α-FtsZ or α-sigma70 antibodies. FtsZ and SigA bands were quantitated using the volume analysis function of the QuantityOne Software. (0.24 MB TIF) [file pone.0011058.s007.tif]

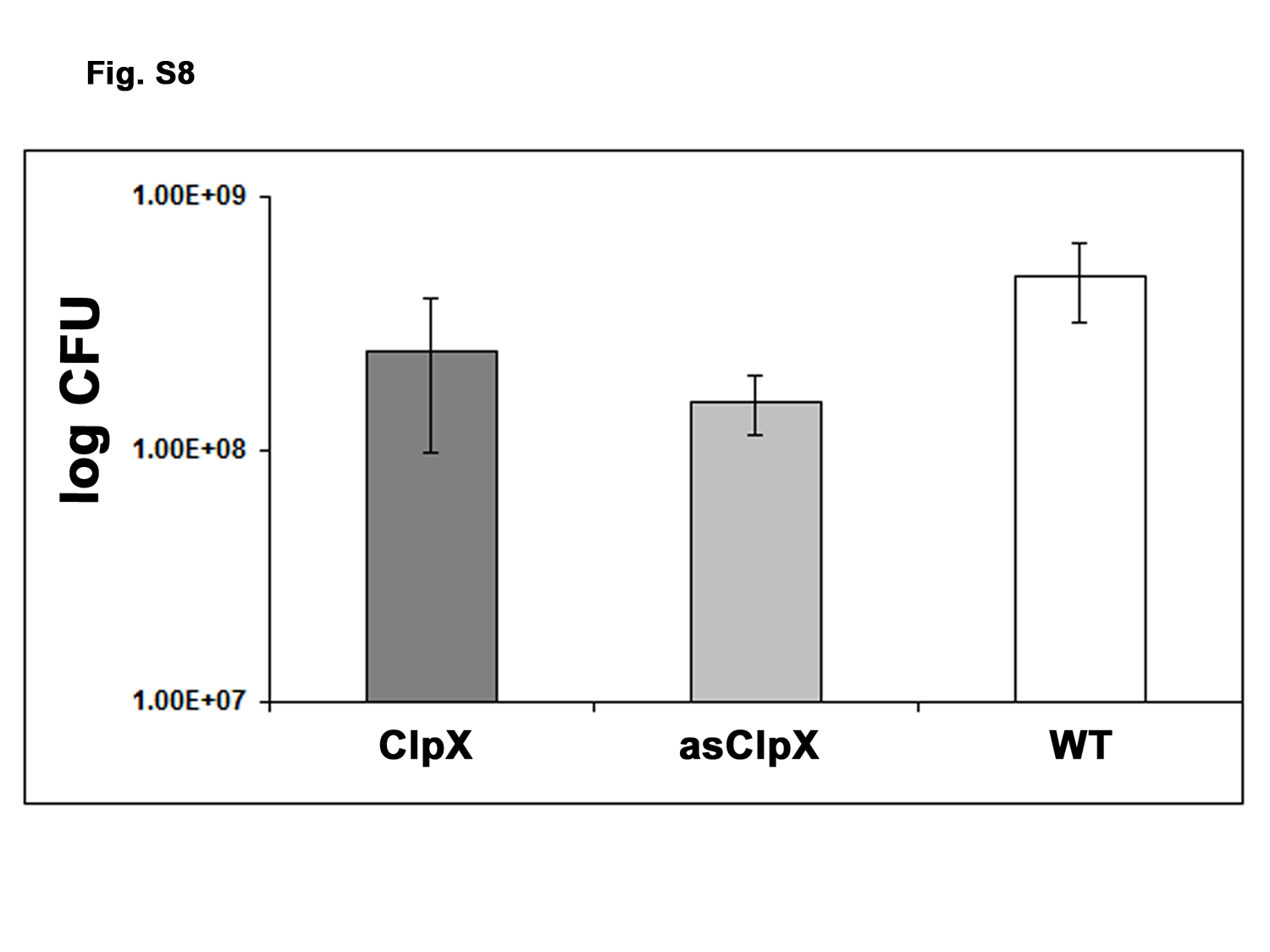

Supplement: Figure S8 — The viability of M. tuberculosis merodiploids overproducing ClpX (ClpX) and depleted in ClpX (asClpX). Broth-grown strains were spread on the plates containing 100 ng/ml anhydrotetracycline. Grown colonies were counted and data plotted using Microsoft Excel. Mean ± SD from three independent experiments are shown. (0.13 MB TIF) [file pone.0011058.s008.tif]
