# Supplementary material for: Mycobacterium tuberculosis ClpX Interacts with FtsZ and Interferes with FtsZ Assembly
Source: PLoS One. 2010 Jul 6;5(7):e11058. doi: 10.1371/journal.pone.0011058 (PMC2897852; doi:10.1371/journal.pone.0011058)
Supplement: Table S1 — (0.07 MB DOC) [file pone.0011058.s009.doc]

**Supplementary Table T1-S.**

| **Cloning primers** | | | |
| --- | --- | --- | --- |
| **Primer name** | **Sequence 5’­­ to 3’** | **Description** | |
| ClpX-PacI | AGAACC**TTAATTAA**GAGCCCCACCAGGGAGGAAGCCGAACGATGGCGCGCATAGGAGACGG | Forward for *clpX* overproduction | |
| ClpX-SwaI | ATCGG**ATTTAAAT**ACTACGCGCTCTTGTCGCGGC | Reverse for sense *clpX* overproduction | |
| asClpX-PacI | AGAACC**TTAATTAA**GAGCCCCACCAGGGAGGAAGCCGAACGCTACGCGCTCTTGTCGCGGC | Forward for antisense *clpX* | |
| asClpX-SwaI | ATCGG**ATTTAAAT**ATGGCGCGCATAGGAGACGG | Reverse for antisense *clpX* | |
| FtsZ-PacI | AGAACC**TTAATTAA**GAGCCCCACCAGGGAGGAAGCC | Forward for *ftsZ* | |
| GFP-SwaI | ATCGG**ATTTAAAT**TATTTGTATAGTTCATCCATGCC | Reverse for *gfp* | |
| FtsZ-21-NdeI | CAGCC**ATATG**ACCCCCCCGCACAAC | Forward for *ftsZ∆C21* in pET19b | |
| FtsZ-21-BamHI | CGC**GGATCC**TCAGTTGGTTGCAACGGCACG | Reverse for *ftsZ∆C21* in pET19b | |
| ClpX-XbaI | CGG**TCTAGA**CGCGCTCTTGTCGCGGC | Reverse for *clpX-gfp fusion* | |
| CFP-XbaI | GC**TCTAGA**AAACAACAACCTGCAGATGGTGAGCAAGGGCGAGGA | Forward for *cfp* | |
| CFP-SwaI | ATCGG**ATTTAAAT**TACTTGTACAGCTCGTCCA | Reverse for *cfp* | |
| FtsZ-YFP-NdeI | CAGC**CATATG**ACCCCCCCGCACAAC | Forward for *ftsZ-yfp* | |
| YFP-ScaI | AAA**AGTACT**TTACTTGTACAGCTCGTCCA | Reverse for *yfp* | |
| DHFR1,2-PacI | AGAACC**TTAATTAA**GAGCCCCACCAGGGAGGAAGCCGAACGATGGTTCGACCATTGAACT | Forward for *dhfr1,2* in pLR52 | |
| DHFR1,2-SwaI | ATCGG**ATTTAAAT**GATCGTACGCTAGTTAACTA | Reverse for *dhfr1,2* in pLR52 | |
| DHFR3-PacI | AGAACC**TTAATTAA**GAGCCCCACCAGGGAGGAAGCCGAACGATGGGCGGAAGTAAAGTAG | Forward for *dhfr3* in pLR56 | |
| DHFR3-SwaI | ATCGG**ATTTAAAT**GTACGCTAGTTAACTACGTC | Reverse for *dhfr3* in pLR56 | |
| ClpX-ClaI | CGATTC**ATCGAT**CGCGCTCTTGTCGCGGCGCTC | Reverse for *clpX* -DHFR1,2 or -DHFR3 in pLR52 and pLR56 | |
| ClpX∆N200-  PacI | AGAACC**TTAATTAA**GAGCCCCACCAGGGAGGAAGCCGAACGATGACTCGCGACGTCTCCGGTGA | Forward for *clpX*∆N200 cloned into DHFR1,2 in pLR52 | |
| FtsZ-ClaI | CGATTC**ATCGAT**GCGGCGCATGAAGGGCGGCA | Reverse for *ftsZ* -DHFR1,2 or -DHFR3 in pLR52 and pLR56 | |
| FtsZ-XbaI | GC**TCTAGA**GATGACCCCCCCGCACAACTACC | Forward for *ftsZ* in pKT25/pUT18C | |
| FtsZ-KpnI | GG**GGTACC**TCAGCGGCGCATGAAGGGCGGC | Reverse for *ftsZ* in pKT25/pUT18C | |
| FtsZ358-KpnI | TTACTTA**GGTACC**CGGTTGGTGTGCAACGGCACGCT | Reverse for *ftsZ∆C21* in pKT25/pUT18 | |
| FsZ-KpnI | CGG**GGTACC**CGGCGGCGCATGAAGGGCGGC | Reverse for *ftsZ* in pKNT25/pUT18 | |
| ClpX-XbaI | GC**TCTAGA**GATGGCGCGCATAGGAGACGG | Forward for *clpX* in pKNT25/pUT18 | |
| ClpX-KpnI | GG**GGTACC**GCGCTCTTGTCGCGGCGCTC | Reverse for *clpX* in pKNT25/pUT18 | |
| ClpX∆N200-XbaI | GC**TCTAGA**GACTCGCGACGTCTCCGGTGAG | Forward for *clpX∆N200* cloned into pKNT25/ pUT18 | |
| FtsQ-BamHI | TCTAGA**GGATCC**CATGACGGAACACAACGAGGA | Forward for *ftsQ*  in pKT25/pUT18C | |
| FtsQ-KpnI | TTACTTA**GGTACC**CGTTTCACGGTCGGCAGGTCG | Reverse for *ftsQ* in pKT25/pUT18C | |
| FtsI-BamHI | TCTAGA**GGATCC**CGTGAGCCGCGCCGCCCCCAG | Forward for *ftsI*  in pKT25/pUT18C | |
| FtsI-EcoRI | CCG**GAATTC**TTAGTTAGGTGGCCTGCAAGACCAAAG | Reverse for *ftsI* in pKT25/pUT18C | |
| FtsQ-NdeI | GGAATTCC**ATATGA**CGGAACACAACGAGG | Forward for *ftsQN100* | |
| FtsQN100-BamHI | CGC**GGATCC**TACTACGCCTTCAGCCCTCGAACAA | Reverse for *ftsQN100* | |
| GCN4-DHFR1,2-PacI | AGAACC**TTAATTAA**GAGCCCCACCAGGGAGGAAGCCGACATATGAACACTGAAGCCGCCAG | M-PFC Forward for *gcn4-dhfr1,2* in pLR52 | |
| GCN4-DHFR1,2-SwaI | ATCGG**ATTTAAAT**CATTAGGTACCCAATTCCGGTTGTTC | M-PFC Reverse for *gcn4-dhfr1,2* in pLR52 | |
| GCN4-DHFR3-PacI | AGAACC**TTAATTAA**GAGCCCCACCAGGGAGGAAGCCGAATTCATGAACACTGAAGCCGCCAG | M-PFC Forward for *gcn4-dhfr3* in pLR56 | |
| GCN4-DHFR3-SwaI | ATCGG**ATTTAAAT**CATTAGTCTTTCTTCTCGTAGACTTC | M-PFC Reverse for *gcn4-dhfr3* in pLR56 | |
| **RT and QRT primers** | | | |
| **Primer name** | **Sequence 5’- 3’** | | **Reference** |
| ClpX-RT | GTTCTCACTCTTGCGGGCGATCTT | | RT primer for *clpX* |
| 16S3-RT | CCGCACGCTCACAG | | RT primer for 16S rRNA |
| asClpX | CTACGCGCTCTTGTCGCGGC | | RT primer for antisense *clpX* |
| ClpX-F | GGCGGTGTACAACCACTACAAGCGGATCCA | | Forward QRT primer for *clpX* |
| ClpX-R | GGCCTGAATAAGTTTGAGCAAGATGTTCTC | | Reverse QRT primer for *clpX* |
| ClpX-TP | CCGGCCTCGGTCAGCGCGGTGGCGTCGGCGA | | TaqMan Probe for *clpX* |
| 16S1 | GAGTGGCGAACGGGTGAGTAACA | | Forward primer for 16S rRNA |
| 16S2 | CACCCCACCAACAAGCTGATAGG | | Reverse primer for 16S rRNA |
| 16S-TP | TCCACCACAAGACATGCATCCCGTG | | TaqMan Probe for 16S rRNA |

**Bold letters** indicate restriction sites**.**
