# Supplementary material for: Mycobacterium tuberculosis ClpX Interacts with FtsZ and Interferes with FtsZ Assembly
Source: PLoS One. 2010 Jul 6;5(7):e11058. doi: 10.1371/journal.pone.0011058 (PMC2897852; doi:10.1371/journal.pone.0011058)
Supplement: Table S2 — (0.11 MB DOC) [file pone.0011058.s010.doc]

**Table S2**

| **Strains** | | |
| --- | --- | --- |
| **Name** | | **Reference** |
| *E. coli* Top10F’ | | Invitrogen Inc. |
| *E. coli* BL21 (DE3) pLysS | | Novagen |
| *E. coli* C41 (DE3) | | Avidis SA, FR |
| *M. tuberculosis* H37Rv | | Lab stock |
| *M. smegmatis* MC^2^155 | | Lab stock |
| **Plasmids** | | |
| **Name** | **Description** | **Reference** |
| pET-19b | *E. coli* expression vector, Amp^r^ | Novagen |
| *pRSFDuet-1* | *E. coli* dual-expression vector, Km^r^ | Novagen |
| pMAL-c4E | *E. coli* expression vector, Amp^r^ | Novagen |
| pMG103 | *E. coli – Mycobacterium* shuttle vector, integrating, with amidase promoter, Km^r^ | This study |
| pLR56 | *E. coli – Mycobacterium* shuttle vector, integrating, with *tet* promoter and tet repressor; Km^r^ | This study |
| pLR52 | *E. coli – Mycobacterium* shuttle vector, replicating, with *tet* promoter and tet repressor, Hyg^r^ | This study |
| pLR66 | *ftsZ* cloned in pRSFDUET-1 in *Nde*I-*Xho*I sites of MCS2, Km^r^ | This study |
| pKT25 | *E. coli* expression vector allowing fusions to C-terminal of the T25 fragment of *cyaA* , Km^r^ | [35] |
| pUT18C | *E. coli* expression vector allowing fusions to C-terminal of the T18 fragment of *cyaA*, Amp^r^ | [35] |
| pKNT25 | *E. coli* expression vector allowing fusions to N-terminal of the T25 fragment of *cyaA* , Km^r^ | [35] |
| pUT18 | *E. coli* expression vector allowing fusions to N-terminal of the T18 fragment of *cyaA*, Amp^r^ | [35] |
| pKT25-zip | Leucine zipper of GCN4 fused to T25 in pKT25 | Euromedex |
| pUT18C-zip | Leucine zipper of GCN4 fused to T18 in pUT18C | Euromedex |
| pUAB100 | *E. coli-Mycobacterium* shuttle vector with  *phsp60::gcn4-dhfr1,2;* replicating, Hyg^r^ | [37] |
| pUAB200 | *E. coli-Mycobacterium* shuttle vector with  *phsp60::gcn4-dhfr3,* integrating, Km^r^ | [37] |
| pRD51 | DHFR [1,2] cloned in pLR52 vector, Hyg^r^ | This study |
| pRD52 | DHFR [3] cloned in pLR56 vector, Km^r^ | This study |
| pSAR1 | *ftsZ_TB_* cloned in pET-19b vector, Amp^r^ | [28] |
| pLR66 | *ftsZ_TB_*  cloned in pRSFDuet-1 vector, Km^r^ | [5] |
| pRR3 | *ftsZ_TB_G103S* cloned in pET-19b vector, Amp^r^ | [28] |
| pRR7 | *ftsZ_TB_D210G* cloned in pET-19b vector, Amp^r^ | [28] |
| pLR12 | *ftsZ_TB_D376A* cloned in pET-19b vector, Amp^r^ | [34] |
| pMK5 | *clpX*_TB_ lacking 1-200 aa from N-terminus (∆N200) cloned in pET-19b, Amp^r^ | This study |
| pMK13 | *ftsZ* lacking 21 aa from C-terminus (∆C21) cloned in pET-19b vector | This study |
| pRD21 | *clpX*_TB_ cloned in pET-19b vector, Amp^r^ | This study |
| pRD3 | *ftsZ*_TB_-*gfp* under the *Ptet* promoter in pLR56, Km^r^ | This study |
| pRD23 | *clpX_TB_* cloned in pLR52, Hyg^r^ | This study |
| pRD33 | Antisense *clpX* sequence cloned in pLR52, Hyg^r^ | This study |
| pRD50 | *clpX_TB_-cfp* in pLR52, Hyg^r^ | This study |
| pMR121 | *ftsZ_TB_-yfp* cloned in pMG103, Km^r^ | This study |
| pRD26 | *ftsQ*_TB_ N-term 1-100 aa (N100) in pET-19b, Amp^r^ | This study |
| pKT25*ftsZ* | *ftsZ_TB_* cloned in pKT25, Km^r^ | This study |
| pKNT25*ftsZ* | *ftsZ_TB_* cloned in pKNT25, Km^r^ | This study |
| pUT18C*ftsZ* | *ftsZ_TB_* cloned in pUT18C, Amp^r^ | This study |
| pUT18*ftsZ* | *ftsZ_TB_* cloned in pUT18, Amp^r^ | This study |
| pKNT25*clpX* | *clpX_TB_* cloned in pKNT25, Km^r^ | This study |
| pUT18*clpX* | *clpX_TB_* cloned in pUT18, Amp^r^ | This study |
| pKNT25*clpX∆N200* | *clpX∆N200_TB_* cloned in pKNT25, Km^r^ | This study |
| pUT18*clpX∆N200* | *clpX∆N200_TB_* cloned in pUT18, Amp^r^ | This study |
| pPP56 | *ftsZ∆C21*_TB_ cloned in pUT18C, Amp^r^ | This study |
| pPP58 | *ftsZG103S*_TB_ cloned in pUT18C, Amp^r^ | This study |
| pPP60 | *ftsZD210G*_TB_ in pUT18C, Amp^r^ | This study |
| pMK18 | *ftsI* cloned in pUT18C, Amp^r^ | This study |
| pMK19 | *ftsI* cloned in pKT25, Km^r^ | This study |
| pMK20 | *ftsQ* cloned in pUT18C, Amp^r^ | This study |
| pMK21 | *ftsQ* cloned in pKT25, Km^r^ | This study |
| pMR118 | *ftsZ*_TB_*- DHFR[3]* cloned in pLR56, Km^r^ | This study |
| pMR119 | *ftsZ*_TB_*- DHFR[1,2]* cloned in pLR52, Hyg^r^ | This study |
| pMK8 | *clpX*_TB_-*DHFR[1,2]* in pLR52, Hyg^r^ | This study |
| pRD53 | *gcn4-DHFR [1,2]* cloned in pLR52, Hyg^r^ | This study |
| pRD54 | *gcn4-DHFR [3]* cloned in pLR56, Km^r^ | This study |
| pRD56 | *clpX∆N200-DHFR[1,2]* cloned in pLR56 | This study |
